# Confidence interval comparison: Precision of maximum likelihood estimates in LLOQ affected data

**DOI:** 10.1371/journal.pone.0293640

**Published:** 2023-11-02

**Authors:** Tanja Bülow, Ralf-Dieter Hilgers, Nicole Heussen

**Affiliations:** 1 Department of Medical Statistics, RWTH Aachen University, Aachen, Germany; 2 Medical School, Sigmund Freud Private University, Vienna, Austria; Medical University of Vienna, AUSTRIA

## Abstract

When data is derived under a single or multiple lower limits of quantification (LLOQ), estimation of distribution parameters as well as precision of these estimates appear to be challenging, as the way to account for unquantifiable observations due to LLOQs needs particular attention. The aim of this investigation is to characterize the precision of censored sample maximum likelihood estimates of the mean for normal, exponential and Poisson distribution affected by one or two LLOQs using confidence intervals (CI).

In a simulation study, asymptotic and bias-corrected accelerated bootstrap CIs for the location parameter mean are compared with respect to coverage proportion and interval width. To enable this examination, we derived analytical expressions of the maximum likelihood location parameter estimate for the assumption of exponentially and Poisson distributed data, where the censored sample method and simple imputation method are used to account for LLOQs. Additionally, we vary the proportion of observations below the LLOQs.

When based on the censored sample estimate, the bootstrap CI led to higher coverage proportions and narrower interval width than the asymptotic CI. The results differed by underlying distribution. Under the assumption of normality, the CI’s coverage proportion and width suffered most from high proportions of unquantifiable observations. For exponentially and Poisson distributed data, both CI approaches delivered similar results. To derive the CIs, the point estimates from the censored sample method are preferable, because the point estimate of the simple imputation method leads to higher bias for all investigated distributions. This biased simple imputation estimate impairs the coverage proportion of the respective CI.

The bootstrap CI surpassed the asymptotic CIs with respect to coverage proportion for the investigated choice of distributional assumptions. The variety of distributions for which the methods are suitable gives the applicant a widely usable tool to handle LLOQ affected data with appropriate approaches.

## Introduction

Every analytical procedure comes with a specific lower limit of quantification (LLOQ), under which the amount of an analyte can not be quantitatively determined with suitable precision and accuracy [1]. An LLOQ of a measurement device will lead to type I left censoring in such data sets [2], consisting of quantifiable and unquantifiable observations. Multiple measurement devices having different LLOQs but delivering data in one study provide the general case of multiple LLOQs.

Methods to estimate the mean and variance of data affected by LLOQs are known in the literature [3]. Recently, Berger *et al.* [4] investigated maximum likelihood estimates for the mean and variance of normally distributed data affected by a single or multiple LLOQs. It was shown that under distributional misspecification, the parameter estimates were strongly biased. With increasing unquantifiable proportion of data, the correct specification of the distribution appeared to be indispensable. Furthermore, they showed that the mean and variance estimates where independently affected by increasing proportions of unquantifiable data. Most existing methods to deal with LLOQ affected data are only applicable in the case of normally distributed data [3–5]. However, exponentially distributed data are worth to be considered e.g. to model growth patterns in human osteosarcoma U2OS cells [6]. Additionally, Poisson distributed data is often found in medical research and application as count data. Similarly, simple imputation methods are widely used in medical research field as well as food regulations. However, numerous publications have shown that those methods generally do not perform well [5, 7, 8] as using one substitution value for all unquantifiable observations will be inadequate. In fact, Helsel [5] even demands that journals should reject papers that use substitution methods. It should be emphasized, that the performance of the SI method will serve as a comparator at how much better the results will be if one uses scientific sound approaches instead of basic ad hoc imputation.

A discussion concerning the quantification of the precision of the estimates is important to obtain a comprehensive understanding of the precision, measured through coverage proportion, and variability, measured as the width of the confidence interval, associated with the estimate of interest. Two commonly used techniques for the construction of confidence intervals are asymptotic, parametrical confidence intervals and non-parametric confidence intervals based on bootstrap procedures.

The paper investigates the performance of confidence intervals in the scenario of multiple LLOQs with increasing proportions of unquantifiable observations and differently distributed data. Therefore, asymptotic and bias-corrected accelerated (BC_*a*_) bootstrap confidence intervals are compared for normally, exponentially and Poisson distributed data with respect to coverage proportion and interval width.

The paper is organized as follows. In the “Methods” section, the estimation model to derive the point estimates is introduced. We selected the method from Berger’s *et al.* [4] analysis with the lowest bias as a basis for the investigation in this paper. This method is derived for the aforementioned distributions. The simple imputation method is introduced as a comparison, as an easily comprehensible and practicable method to handle observations below LLOQs. The investigated confidence intervals are presented in detail and the settings in the simulation study are defined in the sections entitled “Confidence intervals” and “Simulation settings”. In the section entitled “Simulation study”, the results of the simulation study are reported evaluating the performance of the confidence interval types. In the subsequent section entitled “Use case”, a case study using data from Kempf *et al.* [9] is presented and evaluated. The paper concludes with a discussion and gives practical recommendations in the “Discussion” section.

## Methods

To assess the precision of the point estimates from LLOQ affected data, we start by introducing the point estimation model, followed by an introduction of the parametrical, asymptotic and non-parametrical bias-corrected accelerated (BC_*a*_) bootstrap confidence intervals. The estimation model of the censored sample method covers the exponential and Poisson distribution, while the model under normal distribution is introduced by Berger *et al.* [4]. According to the objective of the investigation, two approaches for the computation of confidence intervals are considered. The asymptotic and the bias-corrected accelerated (BC_*a*_) bootstrap confidence intervals will be adopted to the above mentioned distributions and handling methods for LLOQ affected data. At the end of this chapter, the settings for the simulation study are given.

### Estimation model

Let *F* the distribution function and *f* the density function of the random variable W∼F with realizations $w_{k_{i}+j,i}\geq 0$ coming from *m* laboratories with different LLOQs. We define *c*_*i*_ as the *i*−th LLOQ for laboratory *i*, *i* = 1, …, *m* with *c*_*i*_ > *c*_*i*−1_ and *c*_0_ ≔ 0. Let $k_{i}\in N^{0}$ be the number of unquantifiable observations from laboratory *i*, i.e. we assume that the observations *w*_*j*,*i*_ ≥ 0 for *k*_*i*_ > 0 and 1 ≤ *j* ≤ *k*_*i*_ are below *c*_*i*_. It follows that the remaining *n*_*i*_ observations from laboratory *i* are quantifiable, which means *w*_*j*,*i*_ > *c*_*i*_ for all *k*_*i*_ < *j* ≤ *n*_*i*_ + *k*_*i*_. Denote the total number of observations from all laboratories by *N* = *n* + *k* consisting of $n=\sum_{i=1}^{m}n_{i}$ quantifiable and $k=\sum_{i=1}^{m}k_{i}$ unquantifiable observations.

The censored sample method and the simple imputation method are procedures to address unquantified observations due to LLOQ. The censored sample method consists of maximizing the likelihood function assuming censored data, which means that the number of unquantified observations is assumed to be known. According to Berger *et al.* [4] for W∼F the multiple censored sample (CS) method can be written as:

$$\begin{array}{c} L{\left(W\right)}_{F,CS}=\prod_{i=1}^{m}[F{\left(c_{i}\right)}^{k_{i}}\prod_{j=1}^{n_{i}}f(w_{k_{i}+j,i})]. \end{array}$$ (1)

If we apply this model to a normally distributed random variable $X∼N(\mu ,\sigma^{2})$ with $f\left(X;\mu ,\sigma^{2}\right)≔\phi \left(\frac{y-\mu }{\sigma }\right)=\frac{1}{\sigma \sqrt{2\pi }}\text{exp}\{-\frac{{\left(x-\mu \right)}^{2}}{2\sigma^{2}}\}$ as the density function and $F\left(c;\mu ,\sigma^{2}\right)≔\Phi \left(\frac{c-\mu }{\sigma }\right)=\int_{-\infty }^{c}\phi \left(\frac{a-\mu }{\sigma }\right)da$ as the distribution function, the maximum likelihood estimates of the mean ${\hat{\mu }}_{N,CS}$ and variance ${\hat{\sigma }}_{N,CS}^{2}$ can be derived by solving Eqs 2 and 3.

$$\begin{array}{c} 0=\sum_{i=1}^{m}\sum_{j=1}^{n_{i}}x_{k_{i}+j,i}-n\mu -\sigma \sum_{i=1}^{m}k_{i}\frac{\phi (\frac{c_{i}-\mu }{\sigma })}{\Phi (\frac{c_{i}-\mu }{\sigma })}, \end{array}$$ (2)

$$\begin{array}{c} 0=-n\sigma^{2}+\sum_{i=1}^{m}\sum_{j=1}^{n_{i}}{\left(x_{k_{i}+j,i}-\mu \right)}^{2}-\sigma \sum_{i=1}^{m}k_{i}\left(c_{i}-\mu \right)\frac{\phi (\frac{c_{i}-\mu }{\sigma })}{\Phi (\frac{c_{i}-\mu }{\sigma })}. \end{array}$$ (3)

Computationally, the estimates can be derived simultaneously with an appropriate numerical method, like Newton Raphson Method [10]. For the step-by-step derivation of Eqs 2 and 3, see Berger *et al.* [4].

Further, model Eq 1 can be used in the case of exponentially distributed measurements *Y* ∼ *Exp*(λ) for $\lambda \in R_{>0}$ resulting in *F*_λ_(*c*) = 1 − *e*^−*λc*^ as distribution and *f*_λ_(*Y*) = *λe*^−*λy*^, *c*, *x* ≥ 0 as a density function. The likelihood function is represented by Eq 4:

$$\begin{array}{c} L{\left(\lambda ;Y\right)}_{Exp,CS}=\prod_{i=1}^{m}[{\left(1-\text{exp}\{-\lambda c_{i}\}\right)}^{k_{i}}\prod_{j=1}^{n_{i}}\lambda \text{exp}\{-\lambda y_{k_{i}+j,i}\}]. \end{array}$$ (4)

The maximum likelihood estimate ${\hat{\lambda }}_{\text{exp},CS}$ can be computed by numerical solution of Eq 5. The derivation is presented in S1 File.

$$\begin{array}{c} 0=\sum_{i=1}^{m}[\frac{k_{i}c_{i}}{\text{exp}\{\lambda c_{i}\}-1}]+\frac{n}{\lambda }-\sum_{i=1}^{m}\sum_{j=1}^{n_{i}}y_{k_{i}+j,i}. \end{array}$$ (5)

Using Eq 1 for a discrete random variable *Z* ∼ *Poi*(λ) with $f_{\lambda }\left(Z=k\right)=\frac{\lambda^{k}e^{-\lambda }}{k!}$ as the probability mass function and $F_{\lambda }\left(Z=k\right)=e^{-\lambda }\sum_{i=0}^{\left|k\right|}\frac{\lambda^{i}}{i!}$ as the cumulative distribution function, the likelihood function takes the form:

$$\begin{array}{c} L{\left(\lambda ;Z\right)}_{Poi,CS}=\prod_{i=1}^{m}[{\left(\sum_{l=0}^{c_{i}}\text{exp}\{-\lambda \}\frac{\lambda^{l}}{l!}\right)}^{k_{i}}\prod_{j=1}^{n_{i}}\text{exp}\{-\lambda \}\frac{\lambda^{z_{k_{i}+j,i}}}{z_{k_{i}+j,i}!}]. \end{array}$$ (6)

The maximum likelihood estimate ${\hat{\lambda }}_{Poi,CS}$ solving Eq 7 can be computed numerically (see S1 File for the derivation):

$$\begin{array}{c} 0=-n+\frac{1}{\lambda }\sum_{i=1}^{m}\sum_{j=1}^{n_{i}}z_{k_{i}+j,i}-\sum_{i=1}^{m}\frac{k_{i}\text{exp}\left\{-\lambda \right\}\frac{\lambda^{c_{i}}}{c_{i}!}}{\sum_{l=0}^{c_{i}}\text{exp}\left\{-\lambda \right\}\frac{\lambda^{l}}{l!}}. \end{array}$$ (7)

Simple imputation (SI) is a basic but widely used method for the replacement of unquantifiable observations below the LLOQ. Under SI, the unquantified observations are imputed with the imputation value *s* depending on the value of the specific LLOQ *c*_*i*_ for *i* = 1, …, *m*. The most commonly applied choice as imputation value is *s*_*i*_ = *c*_*i*_/2, but also other literature known candidates like $s_{i}\in \{0,c_{i}/\sqrt{2},c_{i}\}$ are without scientific justification. We will use SI wit *s*_*i*_ = *c*_*i*_/2 as a reference method for comparison. The sample mean ${\bar{w}}_{SI(s_{i})}=\frac{1}{N}(\sum_{i=1}^{m}k_{i}s_{i}+\sum_{i=1}^{m}\sum_{j=1}^{n_{i}}w_{k_{i}+j,i})$ and sample variance $s_{SI(s_{i})}^{2}=\frac{1}{N}(\sum_{i=1}^{m}k_{i}{\left(s_{i}-{\hat{\bar{w}}}_{SI(s_{i})}\right)}^{2}+\sum_{i=1}^{m}\sum_{j=1}^{n_{i}}{\left(w_{k_{i}+j,i}-{\hat{\bar{w}}}_{SI(s_{i})}\right)}^{2})$ of a random variable *W* with realizations *w*_*j*,*i*_ form the estimates for the mean and variance of the differently distributed data with ${\hat{\mu }}_{SI(s_{i})}={\bar{w}}_{SI(s_{i})}$ and ${\hat{\sigma }}_{SI(s_{i})}^{2}=s_{SI(s_{i})}^{2}$ for $X∼N(\mu ,\sigma^{2})$, ${\hat{\lambda }}_{SI(s_{i})}=\frac{1}{{\bar{w}}_{SI(s_{i})}}$ for *Y* ∼ *Exp*(λ), and ${\hat{\lambda }}_{SI(s_{i})}={\bar{w}}_{SI(s_{i})}$ for *Z* ∼ *Poi*(λ).

### Confidence intervals

The precision of the point estimates arising from the different methods when dealing with LLOQs of the parameter mean will be analyzed and compared through two different confidence interval (CI) approaches, the parametric confidence interval and the non-parametric bias-corrected accelerated (BC_*a*_) bootstrap method. The first CI approach is the parametrical 95%-CI, as this procedure is widely known and easy to apply by users. The point estimates for mean and variance from the multiple censored sample method or simple imputation method with respective distributional assumptions are used. Based on the estimates that are derived under LLOQs with the above described methodology, the parametrical 95%-CI for E(*W*) for *W* ∈ {*X*, *Y*, *Z*}, will be computed based on the formulae for asymptotic 95%-CI are given in Eq 8 for normal, exponential and Poisson distributed data, respectively [11, 12]:

$$\begin{array}{c} \begin{array}{cc} {95\%\text{-CI}}_{\mu } & =[\hat{\mu }\pm z_{0.975}\frac{\hat{\sigma }}{\sqrt{N}}]\text{for}\;X∼N(\mu ,\sigma^{2}),\\ {95\%\text{-CI}}_{\frac{1}{\lambda }} & =[\frac{2N/\hat{\lambda }}{\chi_{2N;0.975}^{2}};\frac{2N/\hat{\lambda }}{\chi_{2N;0.025}^{2}}]\text{for}\;Y∼Exp(\lambda ),\\ {95\%\text{-CI}}_{\lambda } & =[\hat{\lambda }\pm z_{0.975}\frac{\sqrt{\hat{\lambda }}}{\sqrt{N}}]\text{for}\;Z∼Poi(\lambda ). \end{array} \end{array}$$ (8)

The non-parametric bootstrap bias-corrected accelerated (BC_*a*_) CI by Efron [13] is investigated as an alternative method to construct 95%-CIs for the estimates of the censored sample and simple imputation method. This kind of bootstrap CI is based on bootstrap samples drawn from the LLOQ affected original set. This means, a number of *B* samples are drawn from the original data set with replacement, each of size *N*. These bootstrap samples will include censored data, but not necessary the same censored proportion as in the original data set. Each bootstrap sample will be analyzed with the estimation model presented in the previous section. This will lead to a bootstrap distribution of the estimate for each estimation model respectively. BC_*a*_ intervals use percentiles of the empirical bootstrap distribution, but they do not necessarily use the 100*α*-th and 100(1 − *α*)-th percentiles. They depend on an acceleration parameter $\hat{a}$ and bias-correction factor *z*_0_. The BC_*a*_ level-*α* endpoint follows as ${\hat{\theta }}_{BC_{a}}\left(\alpha \right)=G^{-1}\Phi (z_{0}+\frac{z_{0}+z\left(\alpha \right)}{1-a(z_{0}+z\left(\alpha \right))})$, where *G*(⋅) is defined as the empirical cumulative distribution function of the empirical bootstrap distribution of $\hat{\Phi }$ [13]. With this, the BC_*a*_ bootstrap makes three corrections: through the empirical bootstrap cumulative distribution function of $\hat{\Phi }$, it accounts for the non-normality, through *z*_0_ it accounts for bias, and through *a* for a non-constant error due to a skewed sampling distribution [13–15]. If *z*_0_ and *a* are zero, the BC_*a*_ bootstrap CI reduces to the standard percentile interval. Specifications and derivation of these two parameters are extensively explained in Efron [13]. Although the maximum likelihood estimates from the different methods use distributional assumptions, the procedure to derive the BC_*a*_ bootstrap CI is non-parametric, so the BC_*a*_ CI is considered as a non-parametric confidence interval [14, 16, 17]. When creating the BC_*a*_ bootstrap CI based on the SI method, firstly, the bootstrap replicates are drawn and secondly, each unquantified observation in the bootstrap replicates is imputed following the already explained SI method.

### Simulation settings

In the first part of this simulation study, the appropriate performance of the point estimates is verified to secondly evaluate the behaviour of the parametrical and BC_*a*_ bootstrap CIs.

The code was run on the RWTH Compute Cluster, and written in R version 3.6.1. [18], using packages boot version 1.3–23 [19], censReg version 0.5 and maxLik version 1.3–6 [20, 21]. Function boot.ci(…, type=“bca”) was used to generate the BC_*a*_ bootstrap CI [22].

The results are presented for a fix sample size of *N* = 100. Results for *N* = 40 can be found in the supplementary material S2–S4 Files. For each distribution, the respective data sets were generated as Monte Carlo samples with pre-specified distribution parameters. Simulation runs of *B* = 5500 per scenario were generated, as this number led to stable results regarding the evaluation criteria of the point estimates at the third decimal place. For comparability, we assumed a target censored proportion of observations below the LLOQs resulting in sample dependent LLOQ values. To apply the derived methods, either one LLOQ or two LLOQs are evaluated in the simulation study. To generate the analyzed data set for the case of two LLOQs, *c*_1_ and *c*_2_, the originally drawn random raw data set was split in two halves, where the first *N*/2 observations were censored at *c*_1_ and the second *N*/2 observations at *c*_2_. Unquantified and quantified observations were then merged, taking into account the specific LLOQ for the unquantified observations. To mimic this data generation process, bootstrap replicates were drawn unstratified regarding the belonging LLOQ data set. To generate the BC_*a*_ bootstrap CIs, bootstrap replication number was set to *Rb* = 5500. This bootstrap replication number was chosen as this led to stable results regarding the evaluation criteria of the CIs at the third decimal place. The specific settings can be found in Table 1.

**Table 1:** Simulation settings in simulation study.

distribution	1 LLOQ		2 LLOQs	
	theoretically censored	c_1_	theoretically censored	c_1_;c_2_
N(5.8,1)	0.0%	0	0.0%	0; 0
	20.0%	4.96	20.0%	4.52; 5.28
	50.0%	5.80	50.0%	5.55; 6.05
	65.54%	6.20	65.54%	5.94; 6.49
Exp(0.095)	0.0%	0	0.0%	0; 0
	20.0%	2.35	20.0%	1.11; 375
	50.0%	7.29	50.0%	5.38; 9.65
	65.54%	11.21	65.54%	8.53; 14.82
Poi(4)	0.0%	0	0.0%	0; 0
	23.81%	2	33.58%	2; 3
	43.35%	3	53.11%	3; 4
	62.88%	4	70.70%	4; 5

For each distribution, the respective value of the LLOQ was fixed for the aimed proportion of theoretically censored data.

The point estimates of the multiple censored sample and simple imputation method are vizualized *via* violin plots. Root mean squared error (RMSE), and bias, which is the difference between the true and the estimated parameter, are used for comparison. The root mean squared error is defined as RMSE $=\sqrt{{\left(\bar{\hat{\beta }}-\beta \right)}^{2}+1/\left(B-1\right)\sum_{r=1}^{B}{\left({\hat{\beta }}_{r}-\bar{\hat{\beta }}\right)}^{2}}$, with *β* as the true underlying parameter, and ${\hat{\beta }}_{r}$ as the estimate of this parameter of the *r*-th out of *B* datasets. Precision of the point estimates were assessed by comparing the two types of confidence intervals regarding coverage proportion as well as mean and standard deviation (sd) of the width of the interval estimate.

## Results

### Simulation study

The first step serves to explore the behaviour of the point estimates from the multiple censored sample method and simple imputation method under the distributional assumptions of normal, exponential and Poisson distribution. After this, the results of the precision investigation will be analyzed. The results for two LLOQs are presented in detail. However, the results for the scenario of one LLOQ can be found in S2–S4 Files.

#### Behaviour of point estimates

**Fig 1:**
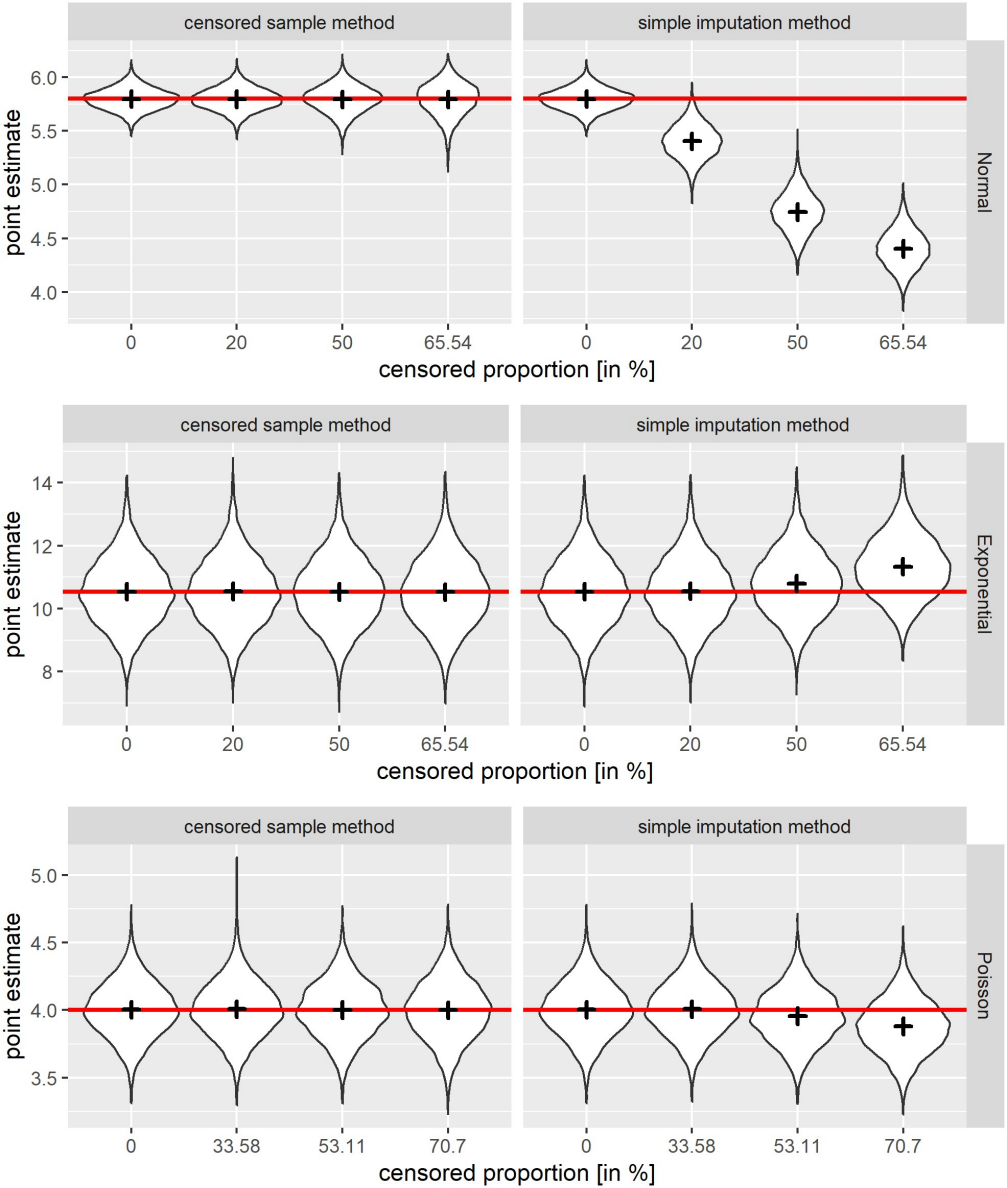
Simulated violin plots of the point estimates for the scenario of 2 LLOQs. Separately for the three different distributional assumptions, with the results of the censored sample method on the left versus simple imputation methods on the right hand side in the scenario of two LLOQs present. Different censored proportions are shown on the x-axis. As a red line, the theoretically underlying parameter mean is presented, indicating estimates closer to the red line as better. The mean of the estimates is shown as a plus. For *B* = 5500, *Rb* = 5500, and *N* = 100.

When evaluating the performance of the point estimates on the basis of the violin plots, the point estimates of the censored sample method clearly outperform the point estimates of the simple imputation method for all presented distributional assumptions, see Fig 1. Comparing the absolut deviation of estimates under censoring compared to no censoring, the point estimate of the censored sample method under exponential and Poisson distribution is not as affected by the increase of censoring as one would suspect from analysing the result under normal distribution. However, also for the simple imputation method, the point estimates of the exponentially and Poisson distributed data are less affected by increased proportion of censored data than of normally distributed data. Also when taking the bias and RMSE as criteria, for all presented distributions, the point estimates of the censored sample method are superior compared to the simple imputation method, see Table A in S2 File. The results in the situation of one LLOQ are similar to that of two LLOQ, also for a sample size of *N* = 40, see S2 File.

#### Confidence interval assessment

**Fig 2:**
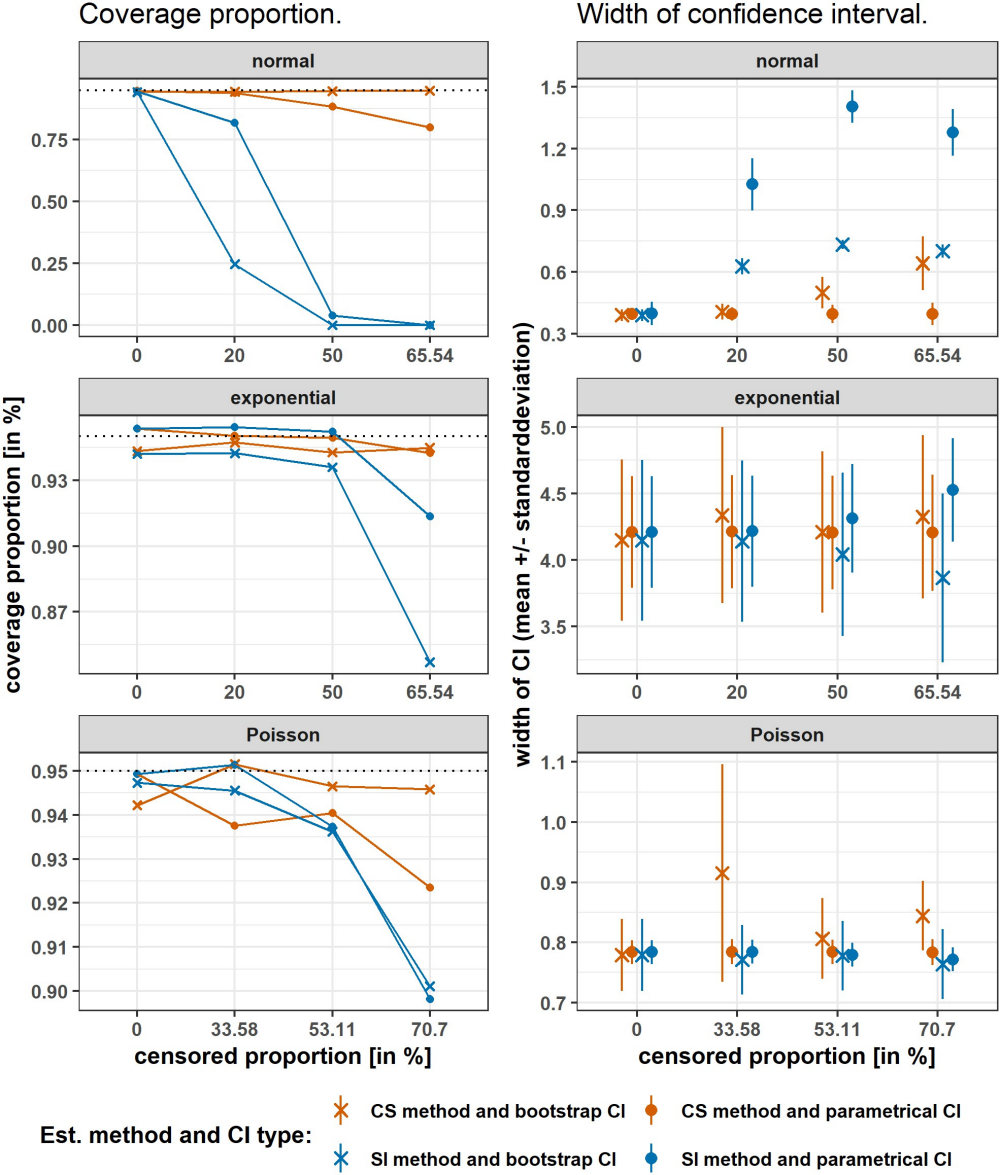
Simulated coverage proportion and mean width of confidence intervals of 2 LLOQs. Separately for the three different distributional assumptions, with the results of the censored sample method (CS) in orange versus simple imputation method (SI) in blue in the scenario of two LLOQs present, results of the BC_*a*_ bootstrap CI marked with an x and of the parametrical CI with a dot. Coverage proportion is shown in the left hand side and width of the CI with mean and standarddeviation on the rigth hand side. Different censored proportions are shown on the x-axis. As a dotted line on the left hand side, the theoretically aimed coverage proportion of 95% is presented, indicating estimates closer to the dotted line as better. For *B* = 5500, *Rb* = 5500, and *N* = 100.

The results in Fig 2 and Table A in S4 File demonstrate that the coverage proportion of both CIs based on the censored sample method are generally much closer to the aimed 0.95 than the CIs based on the simple imputation method. This holds true for all three investigated distributions. Results for one LLOQ and both sample sizes can be found in Figs A-C in S3 File and Table B-D in S4 File. Compared to normally distributed data, the difference between CS and SI based CIs is much smaller up to a censored proportion of 50% for exponentially and Poisson distributed data. Using the censored sample method under the assumption of normal distribution, the BC_*a*_ bootstrap CI stays close to the 95% coverage proportion with increasing amount of censored data. The parametrical CI has the worst coverage proportion of only 0.8007 for the highest amount of censored data. For exponentially distributed data, both CIs using the censored sample method stay above 0.94 for all censored proportions and perform comparably. For Poisson distributed data, both CIs using the censored sample method stay above 0.92 for all censored proportions, but the BC_*a*_ bootstrap CI performs preferably. Using the simple imputation method, the BC_*a*_ bootstrap CI performs worse than the parametrical CI with regard to coverage proportion in all investigated scenarios, see Fig 2. Only for Poisson distributed data, both CIs perform similar with differences in the coverage proportion of maximal 0.58% (Table A in S4 File).

As a second criterion, the width (mean ± sd) of the CI is presented. This criterion should only be seen as a secondary evaluation criterion in case of equal coverage proportions from both CIs. Based on the CS estimate and for the assumption of normality, this is only the case for censoring proportions under or equal to 20%. However, it can be seen in Fig 2 and Table A in S4 File that for a censoring proportion of 20% also the mean width and standard deviation are comparable, 0.4069±0.0378 for the BC_*a*_ bootstrap CI and 0.3953±0.0326 for the parametrical CI. For the other cases, the BC_*a*_ bootstrap CI performs preferably over the parametrical, even if the mean width of the CI is larger. For exponentially distributed data, the coverage proportion is almost equal for all censored amounts of data using the censored sample method. In these cases, the mean width of the parametrical CI is slightly smaller than that of the BC_*a*_ bootstrap CI and for one LLOQ almost equal. Based on the CS estimate and for Poisson distributed data, the mean width of the parametrical CI is smaller than that of the BC_*a*_ bootstrap CI. However, as the coverage proportion of the BC_*a*_ bootstrap CI is better, this is of minor importance. Based on the SI estimate and for Poisson distribution, the BC_*a*_ bootstrap yields a slightly smaller mean width of CI while having nearly equal coverage proportions compared to the parametrical CI.

For a sample size of *N* = 40, these results remain valid in general, refer to S3 and S4 Files. Only for Poisson distribution, the coverage proportion drops to a minimum of around 0.87 for both CIs when based on the censored sample estimate, which is inferior to both CIs when based on the simple imputation method. Having only one LLOQ in the data did not alter the results.

To summarize, we observed that for normally and Poisson distributed data, the BC_*a*_ bootstrap CI based on the censored sample method yielded highest precision of the estimate. For exponentially distributed data, the asymptotic and the BC_*a*_ bootstrap CI reached similar precision when based on the censored sample method.

The computational time to simulate all parametrical confidence intervals was 00:46:23h versus 32:08:02h to simulate all BC_*a*_ bootstrap confidence intervals for a sample size of *N* = 100 on 96 cores of the RWTH High Performance Computing Cluster.

### Use case

We present a case study to illustrate the performance of the parametric and bootstrap CIs using the censored sample and simple imputation method. Table 2 shows the data of 42 randomly selected individual concentration measurements. This data is a subsample taken from a cohort study of employees of a German pharmaceutical company [9]. The uric acid levels measured in *mg*/*dl* from the above-mentioned cohort study are assumed to be normally distributed [23]. Ferritin measured in *ng*/*ml* from the above-mentioned cohort is assumed to be exponentially distributed, whereas the Eosin counts are assumed to be Poisson distributed. To examine the underlying distribution of the three samples, QQ-plots are visually assessed. The QQ-plots for all three data samples (see S1 Fig) show reasonably good behaviour, so that the respective distributional assumptions can be retained. The censored proportion is 54.76% in the uric acid level and Ferritin data and 52.38% in the Eosin data.

**Table 2:** Real example data sets with different distributional assumptions.

distribution	status	observations	number
normal	below *c*_1_	“<5.0”	9
	below *c*_2_	“<5.5”	14
	quantified	5.0, 5.1, 5.7, 5.7, 5.7, 5.8, 5.8, 6.0, 6.1, 6.3,	19
		6.3, 6.4, 6.5, 6.6, 6.7, 6.7, 6.8, 7.0, 7.3	
exponential	below *c*_1_	“<80”	9
	below *c*_2_	“<100”	14
	quantified	84.4, 88.2, 106.0, 108.0, 117.0, 140.0, 144.0,	19
		170.0, 185.0, 209.0, 262.0, 295.0, 103.0,	
		104.0, 106.0, 112.0, 181.0, 280.0, 355.0	
Poisson	below *c*_1_	“<3”	11
	below *c*_2_	“<4”	11
	quantified	3, 3, 3, 3, 3, 3, 4, 4, 5, 5, 5, 6, 6, 6, 6, 6, 7, 7, 8, 12	20

To ensure comparability between the results of Berger *et al.* [4] and this analysis, we decided to take the exact same data example for the normally distributed data. The numerically different result marked with a * (see Table 3) in the point estimate of the censored sample method is due to the fact that the code to solve Eq 1 was improved for this analysis. Here, the log-likelihood functions given in Eqs 2 and 3 were provided as explicit functions, so the numerical algorithm solved these two equations simultaneously. In Berger *et al.* [4], the solving algorithm numerically calculated the log-likelihood function based on the likelihood function. The new procedure used in this manuscript leads to fewer numerically driven artefacts and provides more exact results.

**Table 3:** Point estimates and parametrical and BC_*a*_ bootstrap 95% confidence interval estimates of the real example data sets with different distributional assumptions.

distribution	PE method	PE	CI method	CI estimate	width of CI
normal	censored sample	5.177*	parametrical	[4.823, 5.531]	0.708
			BC_*a*_ bootstrap	[4.613, 5.595]	0.982
	simple imputation	4.250	parametrical	[3.209, 5.291]	2.082
			BC_*a*_ bootstrap	[3.729, 4.806]	1.077
exponential	censored sample	96.218	parametrical	[72.655, 133.504]	60.849
			BC_*a*_ bootstrap	[74.014, 127.412]	53.398
	simple imputation	100.229	parametrical	[75.683, 139.069]	63.386
			BC_*a*_ bootstrap	[80.629, 127.959]	47.330
Poisson	censored sample	3.430	parametrical	[2.870, 3.990]	1.120
			BC_*a*_ bootstrap	[2.769, 4.248]	1.479
	simple imputation	3.417	parametrical	[2.858, 3.976]	1.118
			BC_*a*_ bootstrap	[2.810, 4.179]	1.369

PE: point estimates, CI: confidence interval. The asterisk * marks a numerically different result in the point estimate of the censored sample method compared to Berger *et al.* [4].

Based on the findings from this simulation study and the findings of Berger *et al.* [4], it would be preferable to focus on the point estimates of the censored sample method rather than on the estimates from the simple imputation method. The simulation study shows that for approximately 50% of censored data, the BC_*a*_ bootstrap has better coverage proportions than the parametrical 95%-CI based on the censored sample method. As shown in Table 3 the range of the confidence interval depends on the investigated distributions. The parametrical 95%-CI is smaller for normally and Poisson distributed data, but wider for exponentially distributed data compared to the BC_*a*_ bootstrap.

### Analysis under misspecification

For normal distribution and for both assumptions exponential and Poisson, the parametrical CIs reach higher coverage proportions compared with bootstrap CIs. However, mean width of parametrical CI’s are generally wider than bootstrap CIs (see Fig A in S5 File).

**Fig 3:**
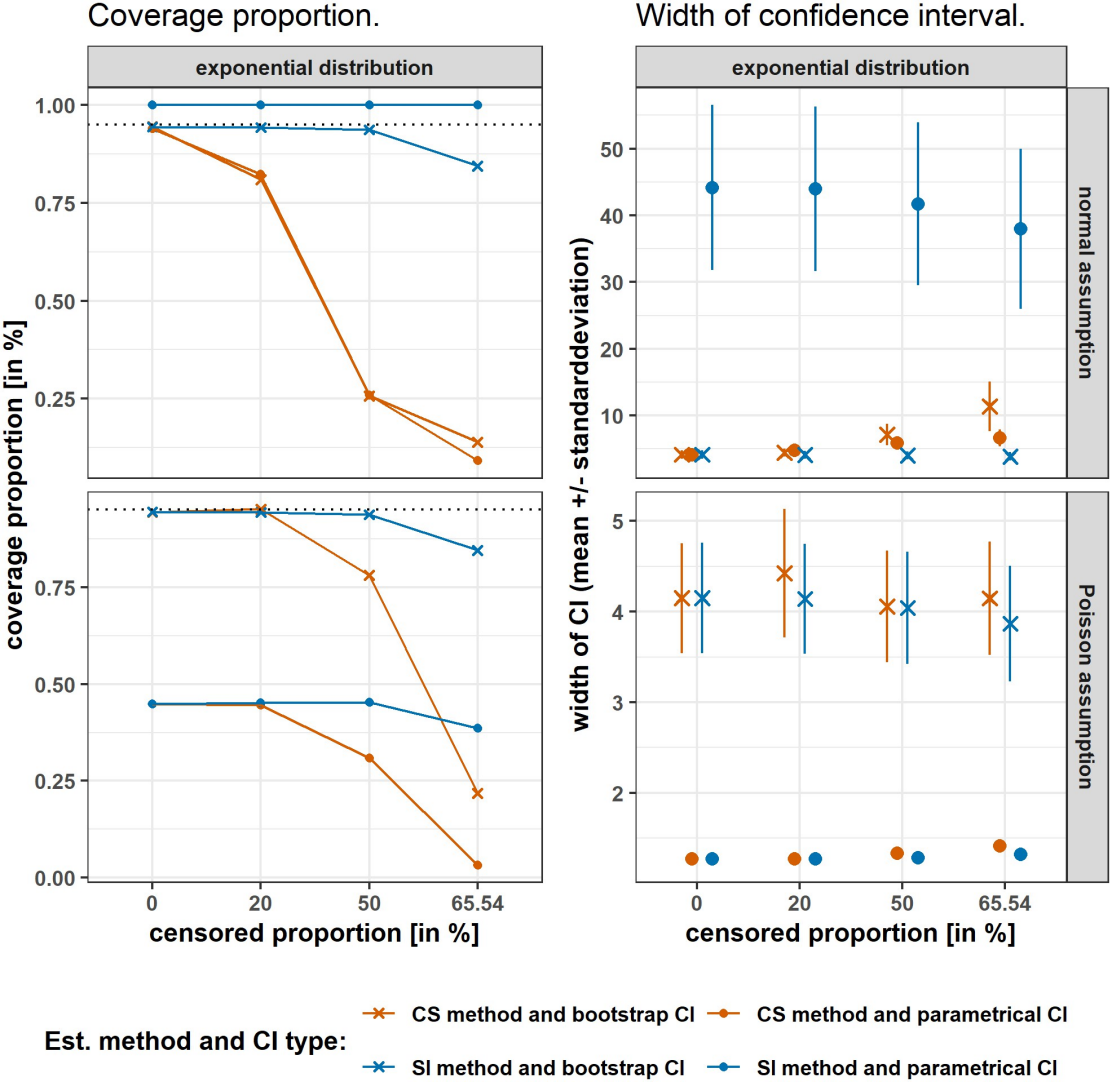
Under assumptional misspecification simulated coverage proportion and mean width of confidence intervals for exponentially distributed data for 2 LLOQs. Performance of both point estimation methods combined with both CIs for exponentially distributed data under respective assumptional misspecification, meaning normal assumption and Poisson assumption. The results of the censored sample method (CS) in orange versus simple imputation method (SI) in blue in the scenario of two LLOQs present, results of the BC_*a*_ bootstrap CI marked with an x and of the parametrical CI with a dot. Coverage proportion is shown in the left hand side and width of the CI with mean and standarddeviation on the rigth hand side. Different censored proportions are shown on the x-axis. As a dotted line on the left hand side, the theoretically aimed coverage proportion of 95% is presented, indicating estimates closer to the dotted line as better. For *B* = 5500, *Rb* = 5500, and *N* = 100.

For exponential distribution but under normal assumption, the SI method combined with bootstrap CI reaches high coverage proportions while maintaining small mean width of CI. The SI method combined with parametrical CI reaches 100% coverage proportion, but the CI is extremely wide, which diminishes the informative value of the CI. Under Poisson assumption, the bootstrap CIs reach relatively high coverage proportion, but again at the cost of wide CIs, see Fig 3.

For Poisson distribution but under normal and exponential assumption, the SI method combined with bootstrap CI reaches high coverage proportions while maintaining small mean width of CI. Under exponential assumption, both bootstrap CIs reach high coverage proportions, but again at the cost of wide CIs (see Fig B in S5 File).

## Discussion

With respect to the two investigated types of confidence intervals, the parametrical and the BC_*a*_ bootstrap, it was shown that both types generated reasonably good estimates for the variability of the point estimates using the censored sample method. This holds true for the case of a single, and two LLOQs present, a proportion of censored data up to 65.54% as well as normally, exponentially and Poisson distributed data. It appeared that both CIs performed similarly well with respect to coverage proportion. Only for normally distributed data, the BC_*a*_ bootstrap was superior.

As demonstrated in the literature [7, 8, 24], the simple imputation method imputing a single value for all missing observations cannot estimate distribution parameters as accurately as parametrical methods. Additionally, both types of investigated CIs perform poorly with regard to coverage proportion and width of CI when using the simple imputation method to obtain the point estimates. This appears to be clear, especially for the parametrical CI, as also the distribution of the data is altered by imputing a single value for all missing observations, whereas this modified distribution is not considered from the parametrical CI. In hypothetical scenarios, an imputation value may be chosen so that the point estimates based on the single imputation method match the underlying true parameter. However, this requires knowing the underlying true parameter, which is unrealistic in almost all practical cases. In conclusion, parametrical methods, such as the censored sample method, should preferably be used to generate point estimates.

The case study illustrates that the point and interval estimates are close to the true value and can be computed and interpreted in a user-friendly way. The confidence intervals differed only marginally between the parametrical and BC_*a*_ bootstrap procedures and gave useful information on the true distribution of the parameter and the variability of the estimate. Besides visually examining a QQ-plot to assess the distributional assumption in a practical case, goodness-of-fit tests, such as Kolmogorov-Smirnov, offer a quantitative approach. However, Fusek [25] shows that the power of this test would only reach 30% in the presented use case. He followed, that such tests should be used for larger sample sizes as the power increases.

The presented approach has several limitations. This publication has focused on a limited set of distributions, which appear to be relevant in real life scenarios. Modelling approaches for the common log-normal distribution have already been studied elsewhere [26–28]. Fusek *et al.* [29] investigated the properties of a maximum likelihood based estimation method under the assumption of Weibull distributed data in the case of single, double or triple censored data. Fusek *et al.* [29] concluded that its use should be favoured over simple imputation methods. Our chosen parametrical confidence intervals did not account for missing values, but assumes complete data. This assumption is violated in the present scenario. We intentionally selected this type of confidence interval as a comparator to contrast this easy and fast approach to the also easy to use but computationally expensive bootstrap procedure. Asymptotically correct parametrical confidence intervals accounting for censored data were developed for normally and log-normally distributed data [16]. No heterogeneity between laboratories is assumed to be present in this investigation. The data from all laboratories was assumed to come from a common distribution. In the case of a violation of this assumption the conclusions drawn here need to be verified. To create the SI based BC_*a*_ bootstrap CI, the order of bootstrapping and imputation followed the recommendation by Shao [30] and Schomaker and Heumann [31], who investigated the combination of bootstrapping and (multiple) imputation. However, as we are using simple imputation with one fix imputation value, only depending on the value of the LLOQ, theoretical consideration indicates that the order will not affect the results. Jeng *et al.* [32] claim for time dependent Type I censoring, recent research indicates that the bootstrap is a powerful procedure for computing accurate approximate confidence intervals especially when using the BC_*a*_ bootstrap procedure. Manly [33] notes that the BC_*a*_ bootstrap confidence interval will give good results for a minimum sample size of 100, when sampling from a normal distribution, but that an even larger sample size is required when sampling from an exponential distribution. This might explain the superior performance of this technique for normally distributed data, but not for the other distributions. Franco-Pereira *et al.* [24] present how to test the discriminatory ability of normally distributed biomarkers, which are subject to an LLOQ based on ROC curve and AUC analysis. Similar to our procedure, they use a parametric approach combined with bootstrap methodology in this scenario. In the context of high-dimensional data, Soret *et al.* [34] presented a Lasso-regularized Buckley-James least squares method in the context of left censored human immunodeficiency virus (HIV) data, but concluded that the parametrical Gaussian Buckley-James method led to most valid results in their investigation. In the case of pharmacokinetic and pharmacodynamic data exposed to LLOQs, Jusko [35] and Keizer *et al.* [8] highlight the ongoing necessity to investigate the handling of such data. However, there is still discussion about how to estimate the LLOQ itself, before an overall analytic strategy for the data can be devised. In a recent article, Wolfinger *et al.* [36] put two approaches up for discussion on the best way to estimate LLOQs for microRNA level. The authors criticise the absence of guidelines on assay quantitation threshold setting for their presented scenario. The 2018 FDA Guidance on Bioanalytical Method Validation [37] now advises to list concentrations below the LLOQ as below the LLOQ, changing their recommendation from the Guidance of 2001 and also deleting a passage in the 2013 Draft FDA validation guidance, where it was stated that “Concentrations below the LLOQ should be reported as zeroes”, see Duggan [38] for comparison. However, a current regulatory recommendation on how to handle LLOQ affected data is missing.

## Conclusion

Our research shows that both investigated confidence interval types give similar information about the variability of the point estimates using the censored sample method. This holds true in the presence of a single or multiple lower limits of quantification for the chosen range of distributional assumptions. As a basis for this investigation, maximum likelihood based censored sample methods to estimate distribution parameters in the case of exponentially or Poisson distributed data were theoretically derived. They prove to have similar properties compared to the corresponding method assuming normal distribution. Researcher can therefore benefit from using the presented procedures if the distribution of the data can be characterised and is partly censored due to LLOQs.

## Supporting information

S1 Fig QQ-plot of the Ferritin use case data. Figure of the QQ-plot of the censored Ferritin use case data to examine if the underlying distribution can be described as exponential.
(PDF)

S1 File Proof of the multiple censored sample method for assumption of exponential and Poisson distribution. (PDF)

S2 File Evaluation of point estimates through violin plot, RMSE, and bias. Corresponding Figures and Tables of the validation of the point estimates for a sample size of *N* = 100 and one LLOQ, and *N* = 40 with one and two LLOQs.
(PDF)

S3 File Evaluation of confidence intervals through coverage proportion and width of CI. Corresponding Figures for the confidence interval asessment for a sample size of *N* = 100 and one LLOQ, and *N* = 40 with one and two LLOQs.
(PDF)

S4 File Tables to evaluate the confidence intervals through coverage proportion and width of CI. Corresponding Tables for the confidence interval asessment for a sample size of *N* = 100 with one and two LLOQs, and *N* = 40 with one and two LLOQs.
(PDF)

S5 File Evaluation of confidence intervals under distributional misspecification through coverage proportion and width of CI. Corresponding Figures for the confidence interval asessment under distributional misspecification for normal and Poisson distribution for a sample size of *N* = 100 and two LLOQs.
(PDF)

S6 File R-code of the simulation study and use case investigation. This code can be used to define the functions, to create the datasets, to generate the figures and tables for the simulation study and to generate the results from the use case.
(ZIP)
