# Supplementary material for: Confidence interval comparison: Precision of maximum likelihood estimates in LLOQ affected data
Source: PLoS One. 2023 Nov 2;18(11):e0293640. doi: 10.1371/journal.pone.0293640 (PMC10621850; doi:10.1371/journal.pone.0293640)
Supplement: S1 Fig — Figure of the QQ-plot of the censored Ferritin use case data to examine if the underlying distribution can be described as exponential. (PDF) [file pone.0293640.s001.pdf]

S1 Figure

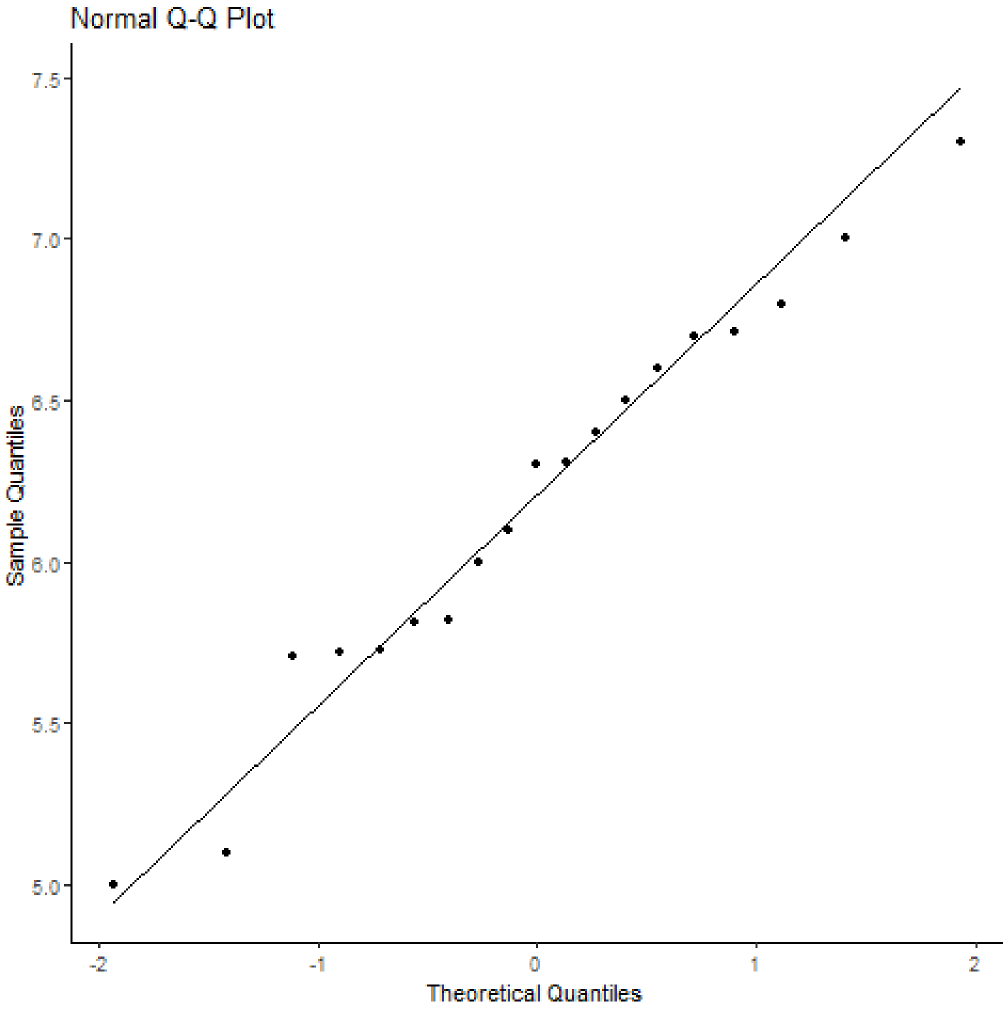

Figure A: **QQ-plot of the censored uric acid use case data to examine if the underlying distribution can be described as normal.**

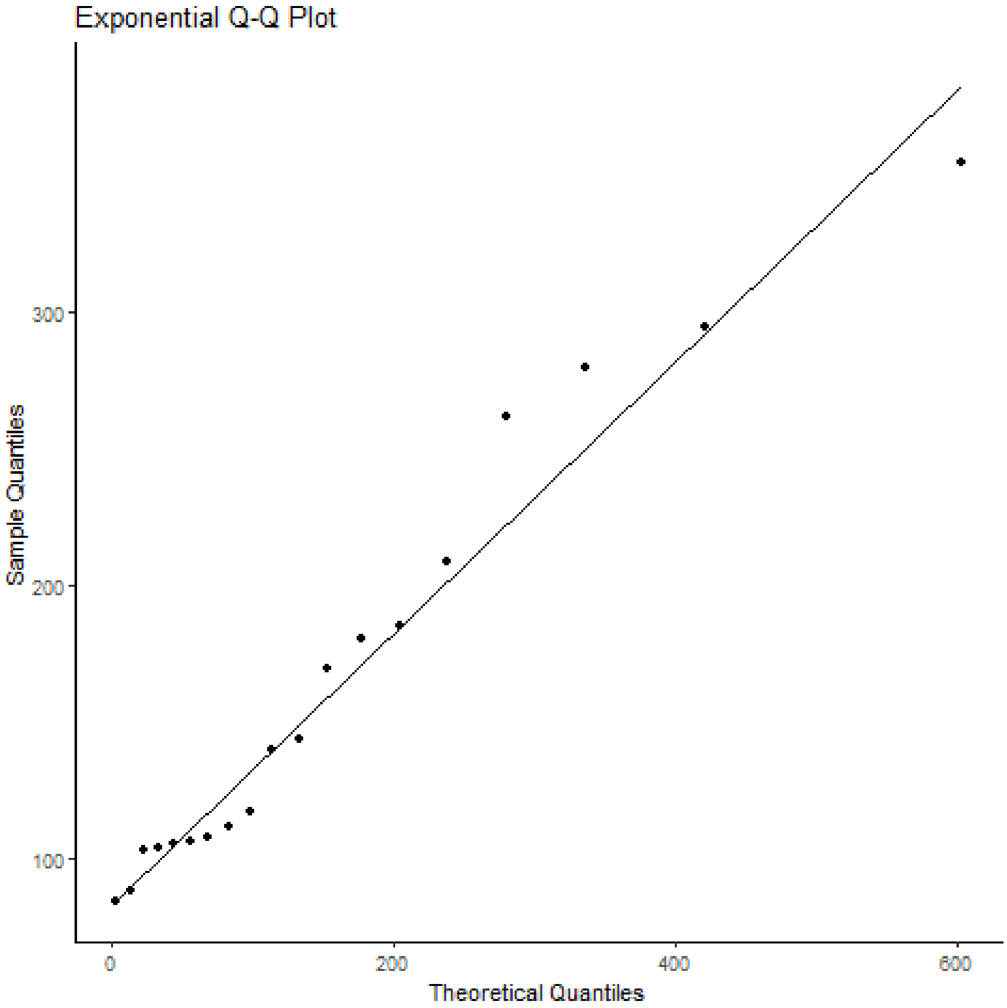

Figure B: **QQ-plot of the censored Ferritin use case data to examine if the underlying distribution can be described as exponential.**

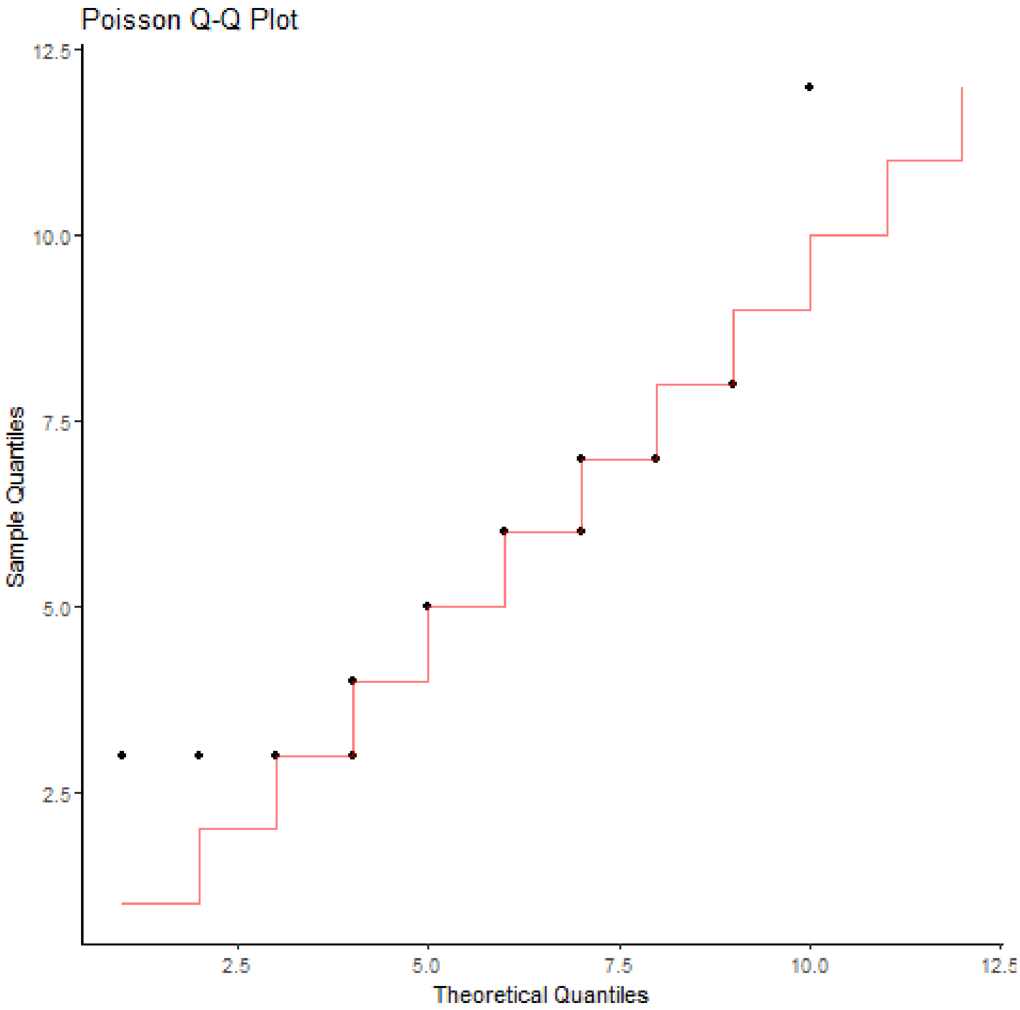

Figure C: **QQ-plot of the censored Eosin use case data to examine if the underlying distribution can be described as Poisson.**
