# Supplementary material for: Confidence interval comparison: Precision of maximum likelihood estimates in LLOQ affected data
Source: PLoS One. 2023 Nov 2;18(11):e0293640. doi: 10.1371/journal.pone.0293640 (PMC10621850; doi:10.1371/journal.pone.0293640)
Supplement: S1 File — (PDF) [file pone.0293640.s002.pdf]

S1 File

Proof of the multiple censored sample method for assumption of exponential and Poisson distribution.

If we assume Eq (5) to be the likelihood function for the assumption of exponentially distributed data, the maximum likelihood estimate from Eq (6) follows as:

$$\begin{aligned}
L(\lambda;Y)_{Exp,CS} &= \prod_{i=1}^{m}\left[(1-\exp\{-\lambda c_i\})^{k_i}\prod_{j=1}^{n_i}\lambda\exp\{-\lambda y_{k_i+j,i}\}\right] \\
l(\lambda;Y)_{Exp,CS} &= \log(L(\lambda;Y)_{Exp,CS}) \\
&= \sum_{i=1}^{m}\log\left((1-\exp\{-\lambda c_i\})^{k_i}\prod_{j=1}^{n_i}\lambda\exp\{-\lambda y_{k_i+j,i}\}\right) \\
&= \sum_{i=1}^{m}k_i\log(1-\exp\{-\lambda c_i\})+\sum_{i=1}^{m}\sum_{j=1}^{n_i}[\log(\lambda)-\lambda y_{k_i+j,i}] \\
&= \sum_{i=1}^{m}k_i\log(1-\exp\{-\lambda c_i\})+n\log(\lambda)-\lambda\sum_{i=1}^{m}\sum_{j=1}^{n_i}y_{k_i+j,i} \\
\frac{\delta l(\lambda;Y)_{Exp,CS}}{\delta\lambda} &= \sum_{i=1}^{m}\left[k_i c_i\frac{\exp\{-\lambda c_i\}}{1-\exp\{-\lambda c_i\}}\right]+\frac{n}{\lambda}-\sum_{i=1}^{m}\sum_{j=1}^{n_i}y_{k_i+j,i} \\
&= \sum_{i=1}^{m}\left[\frac{k_i c_i}{\exp\{\lambda c_i\}-1}\right]+\frac{n}{\lambda}-\sum_{i=1}^{m}\sum_{j=1}^{n_i}y_{k_i+j,i} \\
\frac{\delta l(\lambda;Y)_{Exp,CS}}{\delta\lambda} &= 0\Leftrightarrow 0=\sum_{i=1}^{m}\left[\frac{k_i c_i}{\exp\{\lambda c_i\}-1}\right]+\frac{n}{\lambda}-\sum_{i=1}^{m}\sum_{j=1}^{n_i}y_{k_i+j,i}.
\end{aligned}$$

If we assume Eq (7) to be the likelihood function for the assumption of Poisson distributed data, the maximum likelihood estimate from Eq (8) follows as:

$$\begin{aligned}
L(\lambda;Z)_{Poi,CS} &= \prod_{i=1}^{m}\left[\left(\sum_{l=0}^{c_i}\exp\{-\lambda\}\frac{\lambda^l}{l!}\right)^{k_i}\prod_{j=1}^{n_i}\exp\{-\lambda\}\frac{\lambda^{z_{k_i+j,i}}}{z_{k_i+j,i}!}\right] \\
l(\lambda;Z)_{Poi,CS} &:= \log(L(\lambda;Z)_{Poi,CS}) \\
&= \sum_{i=1}^{m}\Bigg[k_i\log\left(\sum_{l=0}^{c_i}\exp\{-\lambda\}\frac{\lambda^l}{l!}\right) \\
&\quad+\sum_{j=1}^{n_i}(-\lambda+\log(\lambda^{z_{k_i+j,i}})-\log(z_{k_i+j,i}!))\Bigg]
\end{aligned}$$

$$
\begin{aligned}
&= \sum_{i=1}^{m} k_i \log\left(\sum_{l=0}^{c_i} \exp\{-\lambda\}\frac{\lambda^l}{l!}\right) - n\lambda \\
&\quad + \sum_{i=1}^{m}\sum_{j=1}^{n_i} \left(z_{k_i+j,i}\log(\lambda) - \log(z_{k_i+j,i}!)\right) \\
&= \sum_{i=1}^{m} k_i \log\left(\sum_{l=0}^{c_i} \exp\{-\lambda\}\frac{\lambda^l}{l!}\right) - n\lambda \\
&\quad + \log(\lambda)\sum_{i=1}^{m}\sum_{j=1}^{n_i} z_{k_i+j,i} - \sum_{i=1}^{m}\sum_{j=1}^{n_i}\log(z_{k_i+j,i}!) \\
\frac{\delta l(\lambda; Z)_{Poi,CS}}{\delta\lambda} &= -n + \frac{1}{\lambda}\sum_{i=1}^{m}\sum_{j=1}^{n_i} z_{k_i+j,i} \\
&\quad + \sum_{i=1}^{m} k_i \frac{-\sum_{l=0}^{c_i}\exp\{-\lambda\}\frac{\lambda^l}{l!} + \sum_{l=1}^{c_i}\exp\{-\lambda\}\frac{l\lambda^{l-1}}{l!}}{\sum_{l=0}^{c_i}\exp\{-\lambda\}\frac{\lambda^l}{l!}} \\
&= -n + \frac{1}{\lambda}\sum_{i=1}^{m}\sum_{j=1}^{n_i} z_{k_i+j,i} - \sum_{i=1}^{m}\frac{k_i\exp\{-\lambda\}\frac{\lambda^{c_i}}{c_i!}}{\sum_{l=0}^{c_i}\exp\{-\lambda\}\frac{\lambda^l}{l!}} \\
\frac{\delta l(\lambda; Y)_{Poi,CS}}{\delta\lambda} = 0 &\Leftrightarrow 0 = -n + \frac{1}{\lambda}\sum_{i=1}^{m}\sum_{j=1}^{n_i} z_{k_i+j,i} - \sum_{i=1}^{m}\frac{k_i\exp\{-\lambda\}\frac{\lambda^{c_i}}{c_i!}}{\sum_{l=0}^{c_i}\exp\{-\lambda\}\frac{\lambda^l}{l!}}.
\end{aligned}
$$
