# Supplementary material for: Confidence interval comparison: Precision of maximum likelihood estimates in LLOQ affected data
Source: PLoS One. 2023 Nov 2;18(11):e0293640. doi: 10.1371/journal.pone.0293640 (PMC10621850; doi:10.1371/journal.pone.0293640)
Supplement: S2 File — Corresponding Figures and Tables of the validation of the point estimates for a sample size of N = 100 and one LLOQ, and N = 40 with one and two LLOQs. (PDF) [file pone.0293640.s003.pdf]

S2 File

Evaluation of point estimates through violin plot, RMSE, and bias.

For N=100, 2 LLOQ.

Table A: Evaluation of point estimates through RMSE and bias. 2 LLOQs, $B = 5500$, $Rb = 5500$, $n = 100$.

distribution	estimation method	censored prop. in %	RMSE	bias
normal	censored sample method	0.00	0.10	−0.00
		20.00	0.10	−0.00
		50.00	0.13	−0.01
		65.54	0.16	−0.01
	simple imputation method	0.00	0.10	−0.00
		20.00	0.43	−0.40
		50.00	1.07	−1.05
		65.54	1.41	−1.40
exponential	censored sample method	0.00	1.05	0.01
		20.00	1.06	0.02
		50.00	1.07	0.01
		65.54	1.09	0.00
	simple imputation method	0.00	1.05	0.01
		20.00	1.05	0.03
		50.00	1.06	0.27
		65.54	1.26	0.81
Poisson	censored sample method	0.00	0.20	0.00
		33.58	0.21	0.01
		53.11	0.21	0.00
		70.70	0.22	−0.00
	simple imputation method	0.00	0.20	0.00
		33.58	0.20	0.01
		53.11	0.20	−0.04
		70.70	0.23	−0.12

For N=100, 1 LLOQ.

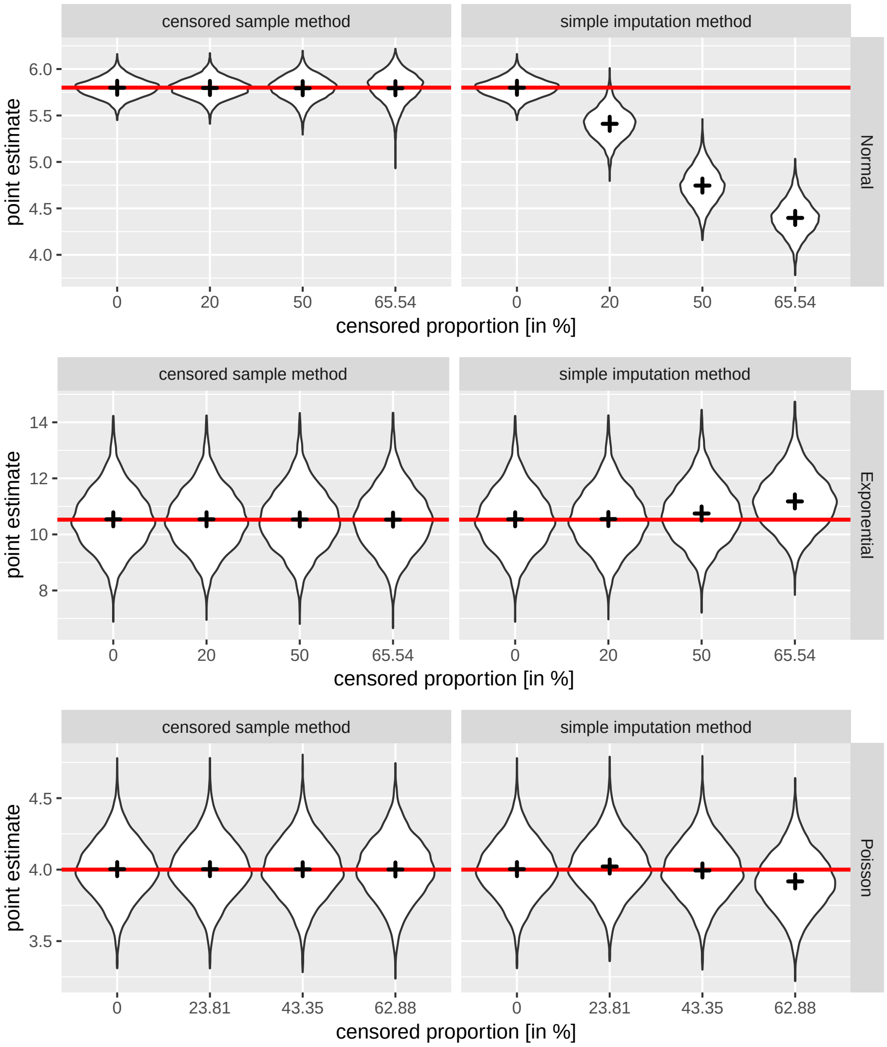

Figure A: **Simulated violin plots of the point estimates for the scenario of $N = 100$ and 1 LLOQ.** Separately for the three different distributional assumptions, with the results of the censored sample method on the left versus simple imputation methods on the right hand side in the scenario of one LLOQ present. Different censored proportions are shown on the x-axis. As a red line, the theoretically underlying parameter mean is presented, indicating estimates closer to the red line as better. The mean of the estimates is shown as a plus. For $B = 5500$, $Rb = 5500$, and $N = 100$.

Table B: Evaluation of point estimates through RMSE and bias. 1 LLOQ, $B = 5500$, $Rb = 5500$, $n = 100$.

distribution	estimation method	censored prop. in %	RMSE	bias
normal	censored sample method	0.00	0.10	-0.00
		20.00	0.10	-0.00
		50.00	0.13	-0.01
		65.54	0.16	-0.01
	simple imputation method	0.00	0.10	−0.00
		20.00	0.42	−0.39
		50.00	1.07	−1.05
		65.54	1.41	−1.40
exponential	censored sample method	0.00	1.05	0.01
		20.00	1.05	0.01
		50.00	1.06	0.01
		65.54	1.09	0.00
	simple imputation method	0.00	1.05	0.01
		20.00	1.05	0.02
		50.00	1.05	0.22
		65.54	1.18	0.65
Poisson	censored sample method	0.00	0.20	0.00
		23.81	0.20	0.00
		43.35	0.20	0.00
		62.88	0.21	0.00
	simple imputation method	0.00	0.20	0.00
		23.81	0.20	0.02
		43.35	0.20	−0.01
		62.88	0.22	−0.08

For N=40, 2 LLOQ.

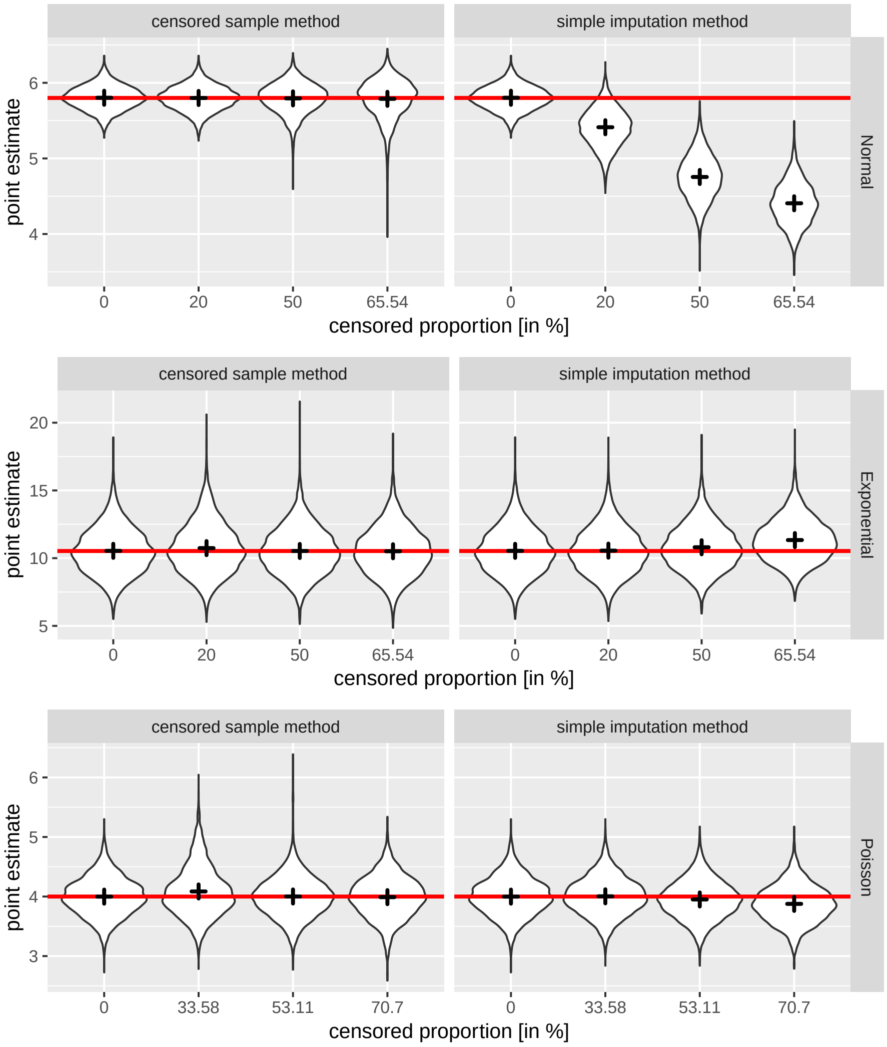

Figure B: **Simulated violin plots of the point estimates for the scenario of $N = 40$ and 2 LLOQ.** Separately for the three different distributional assumptions, with the results of the censored sample method on the left versus simple imputation methods on the right hand side in the scenario of two LLOQ present. Different censored proportions are shown on the x-axis. As a red line, the theoretically underlying parameter mean is presented, indicating estimates closer to the red line as better. The mean of the estimates is shown as a plus. For $B = 5500$, $Rb = 5500$, and $N = 40$.

Table C: Evaluation of point estimates through RMSE and bias. 2 LLOQ, $B = 5500$, $Rb = 5500$, $n = 40$.

distribution	estimation method	censored prop. in %	RMSE	bias
normal	censored sample method	0.00	0.16	0.00
		20.00	0.16	−0.00
		50.00	0.20	−0.01
		65.54	0.26	−0.01
	simple imputation method	0.00	0.16	0.00
		20.00	0.46	−0.39
		50.00	1.09	−1.05
		65.54	1.42	−1.39
exponential	censored sample method	0.00	1.70	0.01
		20.00	1.88	0.21
		50.00	1.72	0.00
		65.54	1.76	-0.02
	simple imputation method	0.00	1.70	0.01
		20.00	1.70	0.03
		50.00	1.67	0.28
		65.54	1.76	0.81
Poisson	censored sample method	0.00	0.32	-0.00
		33.58	0.42	0.09
		53.11	0.35	0.00
		70.70	0.35	-0.01
	simple imputation method	0.00	0.32	-0.00
		33.58	0.32	0.00
		53.11	0.32	-0.05
		70.70	0.34	-0.12

For N=40, 1 LLOQ.

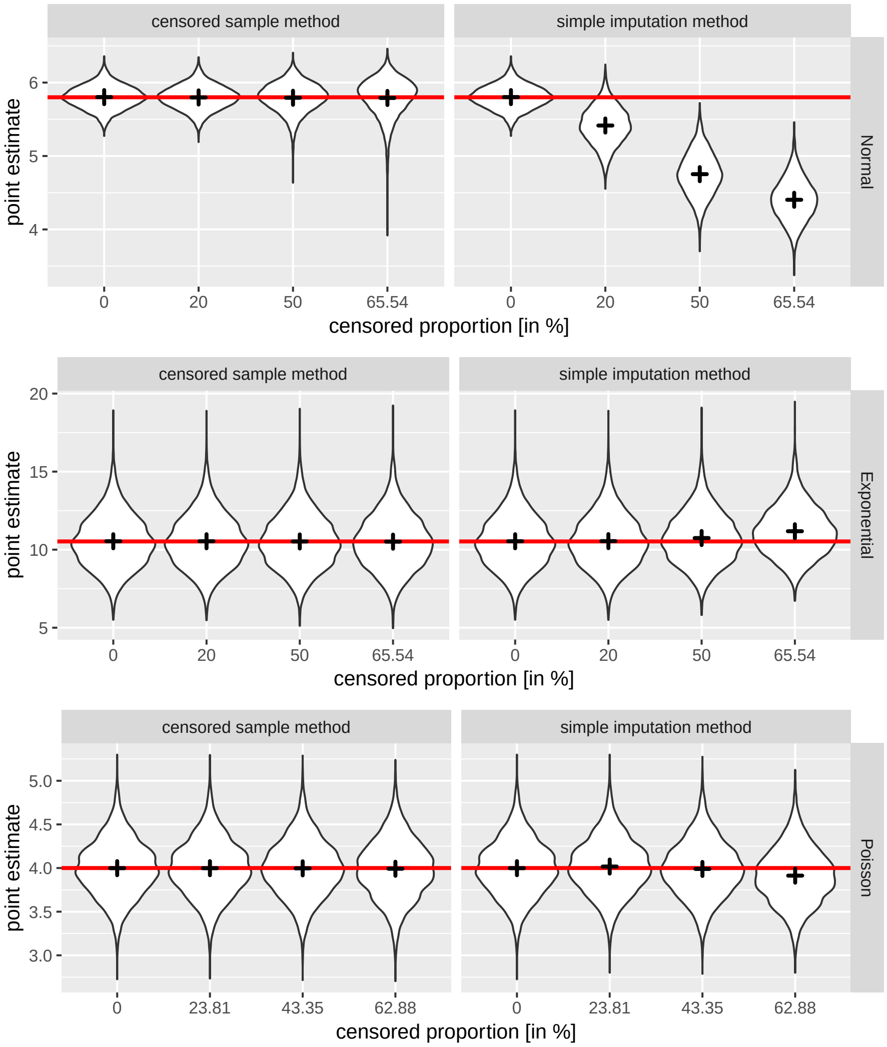

Figure C: **Simulated violin plots of the point estimates for the scenario of $N = 40$ and 1 LLOQ.** Separately for the three different distributional assumptions, with the results of the censored sample method on the left versus simple imputation methods on the right hand side in the scenario of one LLOQ present. Different censored proportions are shown on the x-axis. As a red line, the theoretically underlying parameter mean is presented, indicating estimates closer to the red line as better. The mean of the estimates is shown as a plus. For $B = 5500$, $Rb = 5500$, and $N = 40$.

Table D: Evaluation of point estimates through RMSE and bias. 1 LLOQ, $B = 5500$, $Rb = 5500$, $n = 40$.

distribution	estimation method	censored prop. in %	RMSE	bias
normal	censored sample method	0.00	0.16	0.00
		20.00	0.16	-0.00
		50.00	0.20	-0.01
		65.54	0.26	-0.01
	simple imputation method	0.00	0.16	0.00
		20.00	0.46	-0.38
		50.00	1.09	-1.05
		65.54	1.42	-1.40
exponential	censored sample method	0.00	1.70	0.01
		20.00	1.70	0.01
		50.00	1.72	-0.00
		65.54	1.75	-0.01
	simple imputation method	0.00	1.70	0.01
		20.00	1.70	0.02
		50.00	1.67	0.22
		65.54	1.71	0.65
Poisson	censored sample method	0.00	0.32	-0.00
		23.81	0.32	-0.00
		43.35	0.32	-0.00
		62.88	0.34	-0.01
	simple imputation method	0.00	0.32	-0.00
		23.81	0.31	0.02
		43.35	0.32	-0.01
		62.88	0.33	-0.09
