# Supplementary material for: Confidence interval comparison: Precision of maximum likelihood estimates in LLOQ affected data
Source: PLoS One. 2023 Nov 2;18(11):e0293640. doi: 10.1371/journal.pone.0293640 (PMC10621850; doi:10.1371/journal.pone.0293640)
Supplement: S3 File — Corresponding Figures for the confidence interval asessment for a sample size of N = 100 and one LLOQ, and N = 40 with one and two LLOQs. (PDF) [file pone.0293640.s004.pdf]

S3 File

Evaluation of confidence intervals through coverage proportion and width of CI.

For N=100, 1 LLOQ.

For N=40, 2 LLOQ.

For N=40, 1 LLOQ.

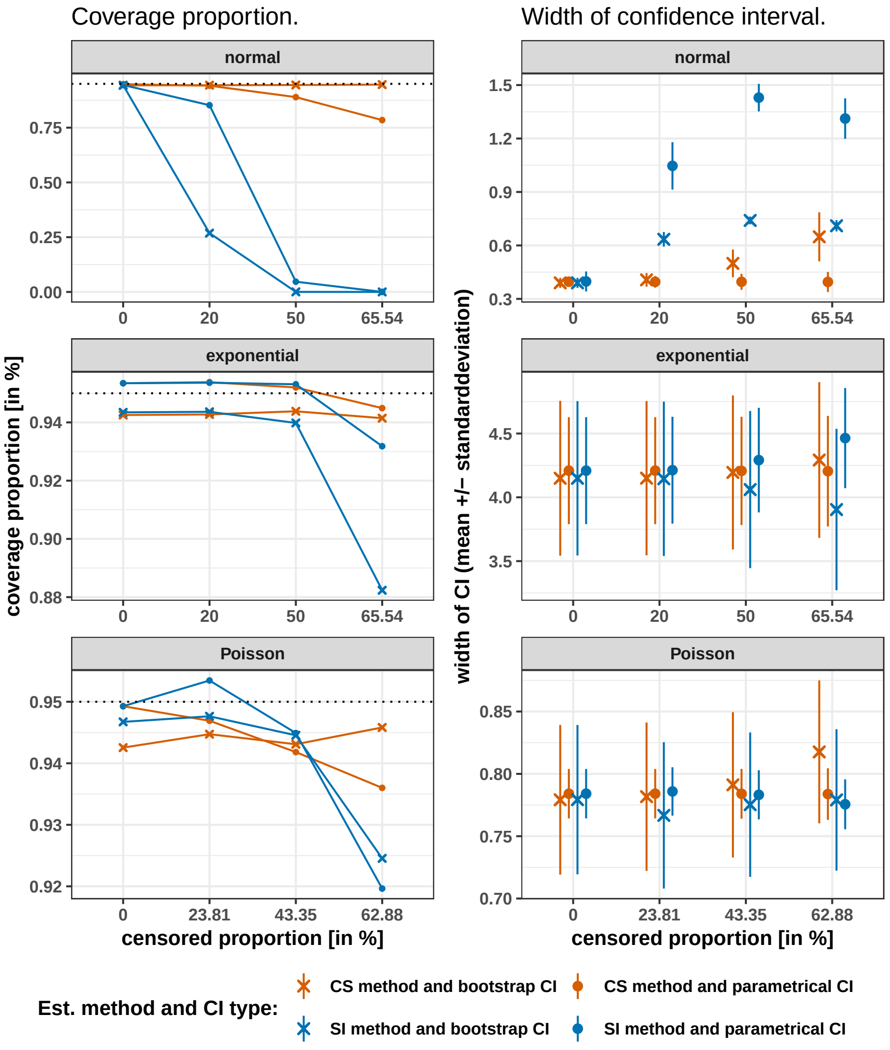

Figure A: **Simulated coverage proportion and mean width of confidence intervals 1 LLOQs.** Separately for the three different distributional assumptions, with the results of the censored sample method (CS) in orange versus simple imputation method (SI) in blue in the scenario of two LLOQs present, results of the BC_a bootstrap CI marked with an x and of the parametrical CI with a dot. Coverage proportion is shown in the left hand side and width of the CI with mean and standarddeviation on the rigth hand side. Different censored proportions are shown on the x-axis. As a dotted line on the left hand side, the theoretically underlying parameter mean is presented, indicating estimates closer to the dotted line as better. For 1 LLOQ, $B = 5500$, $Rb = 5500$, and $N = 100$.

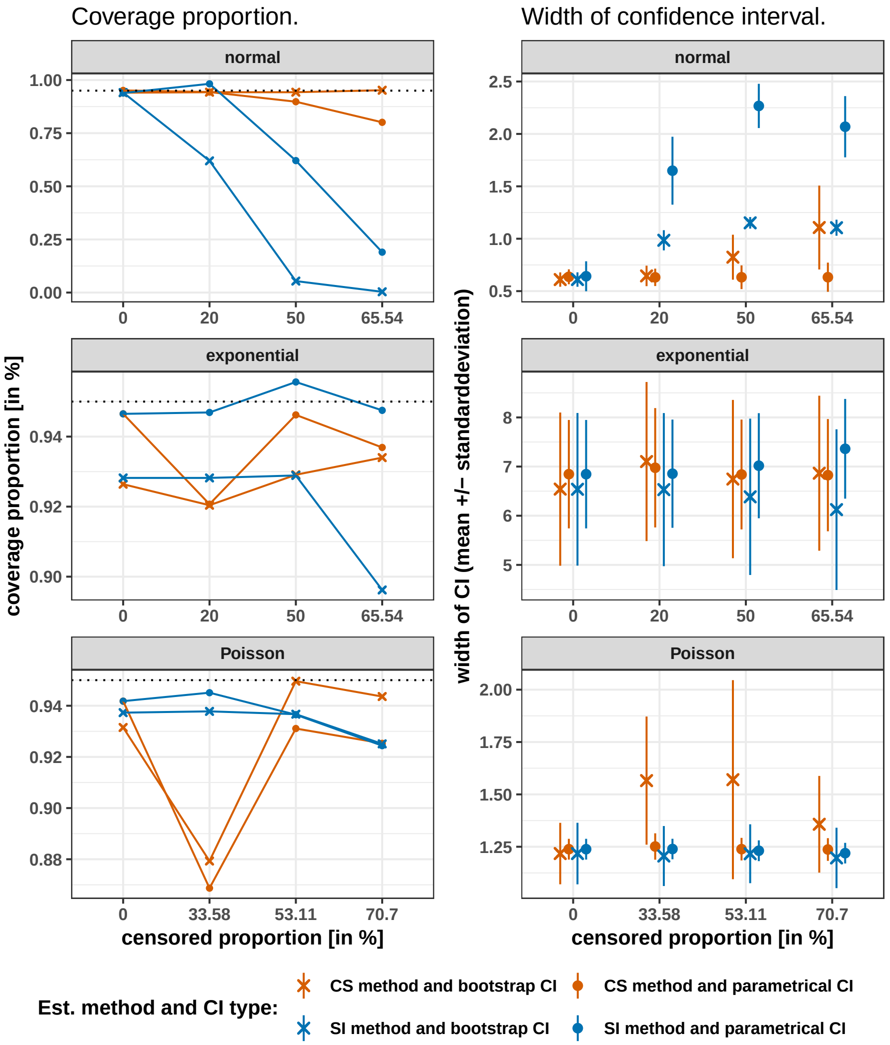

Figure B: **Simulated coverage proportion and mean width of confidence intervals 2 LLOQs.** Separately for the three different distributional assumptions, with the results of the censored sample method (CS) in orange versus simple imputation method (SI) in blue in the scenario of two LLOQs present, results of the BC_a bootstrap CI marked with an x and of the parametrical CI with a dot. Coverage proportion is shown in the left hand side and width of the CI with mean and standarddeviation on the rigth hand side. Different censored proportions are shown on the x-axis. As a dotted line on the left hand side, the theoretically underlying parameter mean is presented, indicating estimates closer to the dotted line as better. For 2 LLOQs, $B = 5500$, $Rb = 5500$, and $N = 40$.

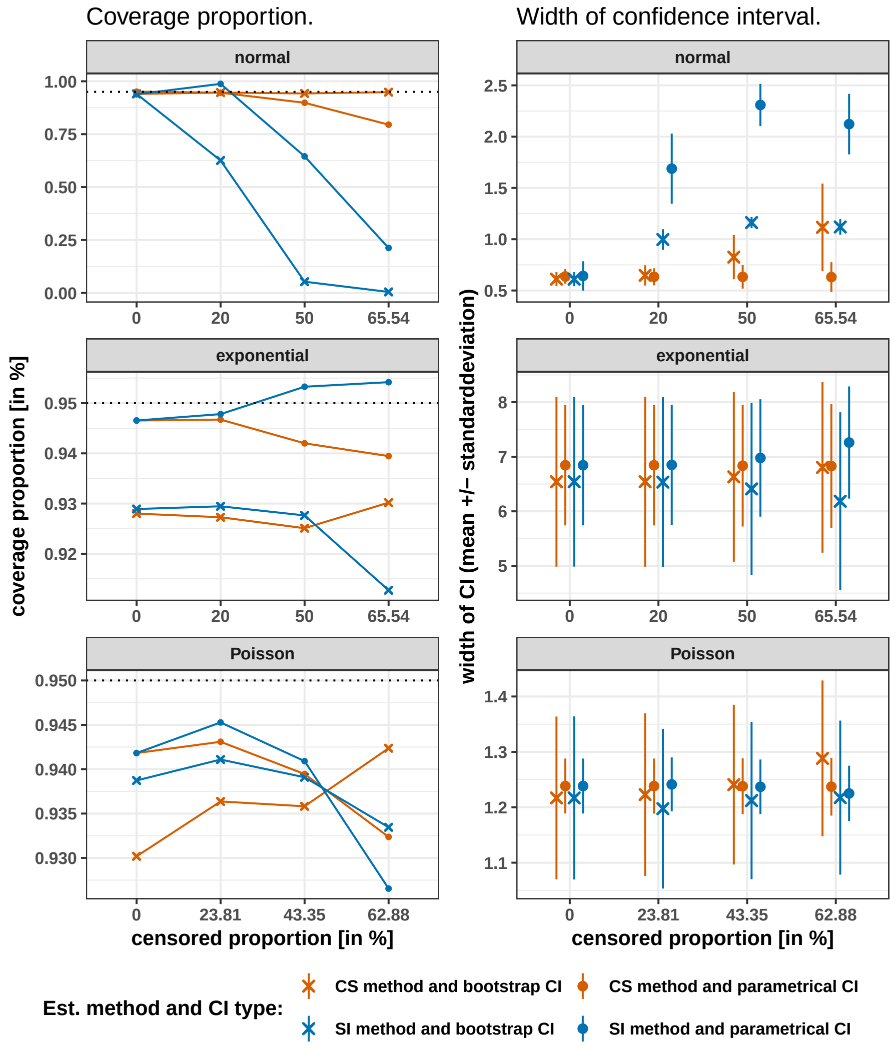

Figure C: **Simulated coverage proportion and mean width of confidence intervals 1 LLOQs.** Separately for the three different distributional assumptions, with the results of the censored sample method (CS) in orange versus simple imputation method (SI) in blue in the scenario of two LLOQs present, results of the BC_a bootstrap CI marked with an x and of the parametrical CI with a dot. Coverage proportion is shown in the left hand side and width of the CI with mean and standarddeviation on the rigth hand side. Different censored proportions are shown on the x-axis. As a dotted line on the left hand side, the theoretically underlying parameter mean is presented, indicating estimates closer to the dotted line as better. For 1 LLOQ, $B = 5500$, $Rb = 5500$, and $N = 40$.
