# Supplementary material for: Confidence interval comparison: Precision of maximum likelihood estimates in LLOQ affected data
Source: PLoS One. 2023 Nov 2;18(11):e0293640. doi: 10.1371/journal.pone.0293640 (PMC10621850; doi:10.1371/journal.pone.0293640)
Supplement: S4 File — Corresponding Tables for the confidence interval asessment for a sample size of N = 100 with one and two LLOQs, and N = 40 with one and two LLOQs. (PDF) [file pone.0293640.s005.pdf]

Tables for coverage proportion and mean width of confidence interval

2 LLOQ, N=100

1 LLOQ, N=100

2 LLOQ, N=40

1 LLOQ, N=40

Table A: Evaluation of confidence intervals for 2 LLOQs.

distribution	estimation method	censored prop.	CI type	coverage prop.	mean width of CI (sd)
normal	censored sample method	0.00%	BC$_a$ bootstrap	0.9436	0.3899 (0.0280)
			parametrical	0.9485	0.3964 (0.0280)
		20.00%	BC$_a$ bootstrap	0.9438	0.4069 (0.0378)
			parametrical	0.9400	0.3953 (0.0326)
		50.00%	BC$_a$ bootstrap	0.9471	0.4989 (0.0755)
			parametrical	0.8847	0.3956 (0.0440)
		65.54%	BC$_a$ bootstrap	0.9485	0.6422 (0.1301)
			parametrical	0.8007	0.3950 (0.0539)
	simple imputation method	0.00%	BC$_a$ bootstrap	0.9433	0.3900 (0.0280)
			parametrical	0.9447	0.3979 (0.0561)
		20.00%	BC$_a$ bootstrap	0.2465	0.6279 (0.0394)
			parametrical	0.8193	1.0262 (0.1270)
		50.00%	BC$_a$ bootstrap	0.0000	0.7337 (0.0228)
			parametrical	0.0395	1.4044 (0.0791)
		65.54%	BC$_a$ bootstrap	0.0000	0.7014 (0.0319)
			parametrical	0.0000	1.2786 (0.1128)
exponential	censored sample method	0.00%	BC$_a$ bootstrap	0.9433	4.1496 (0.6065)
			parametrical	0.9535	4.2093 (0.4192)
		20.00%	BC$_a$ bootstrap	0.9473	4.3367 (0.6614)
			parametrical	0.9504	4.2126 (0.4246)
		50.00%	BC$_a$ bootstrap	0.9427	4.2091 (0.6055)
			parametrical	0.9493	4.2062 (0.4261)
		65.54%	BC$_a$ bootstrap	0.9449	4.3239 (0.6143)
			parametrical	0.9425	4.2048 (0.4368)
	simple imputation method	0.00%	BC$_a$ bootstrap	0.9420	4.1477 (0.6037)
			parametrical	0.9535	4.2089 (0.4193)
		20.00%	BC$_a$ bootstrap	0.9424	4.1404 (0.6074)
			parametrical	0.9542	4.2161 (0.4183)
		50.00%	BC$_a$ bootstrap	0.9360	4.0417 (0.6152)
			parametrical	0.9522	4.3132 (0.4087)
		65.54%	BC$_a$ bootstrap	0.8471	3.8658 (0.6344)
			parametrical	0.9135	4.5262 (0.3889)
Poisson	censored sample method	0.00%	BC$_a$ bootstrap	0.9422	0.7792 (0.0596)
			parametrical	0.9493	0.7841 (0.0198)
		33.58%	BC$_a$ bootstrap	0.9515	0.9151 (0.1811)
			parametrical	0.9376	0.7845 (0.0209)
		53.11%	BC$_a$ bootstrap	0.9465	0.8063 (0.0671)
			parametrical	0.9404	0.7839 (0.0203)
		70.70%	BC$_a$ bootstrap	0.9458	0.8441 (0.0577)
			parametrical	0.9235	0.7837 (0.0214)
	simple imputation method	0.00%	BC$_a$ bootstrap	0.9473	0.7793 (0.0598)
			parametrical	0.9493	0.7841 (0.0198)
		33.58%	BC$_a$ bootstrap	0.9455	0.7713(0.0579)
			parametrical	0.9513	0.7845 (0.0196)
		53.11%	BC$_a$ bootstrap	0.9362	0.7779 (0.0575)
			parametrical	0.9373	0.7794 (0.0197)
		70.70%	BC$_a$ bootstrap	0.9011	0.7641 (0.0583)
			parametrical	0.8982	0.7719 (0.0197)

CI: confidence interval, prop.: proportion, sd: standard deviation. Simulation runs $B = 5500$, bootstrap replication number $Rb = 5500$, sample size was $N = 100$, 2 LLOQ.

Table B: **Evaluation of confidence intervals for 1 LLOQ,** $N = 100$**.**

distribution	estimation method	censored prop.	CI type	coverage prop.	mean width of CI (sd)
normal	censored sample method	0.00%	BC_a bootstrap	0.9431	0.3900 (0.0281)
			parametrical	0.9485	0.3964 (0.0280)
		20.00%	BC_a bootstrap	0.9438	0.4067 (0.0380)
			parametrical	0.9416	0.3957 (0.0327)
		50.00%	BC_a bootstrap	0.9447	0.4991 (0.0776)
			parametrical	0.8895	0.3958 (0.0449)
		65.54%	BC_a bootstrap	0.9465	0.6484 (0.1379)
			parametrical	0.7845	0.3951 (0.0561)
	simple imputation method	0.00%	BC_a bootstrap	0.9438	0.3898 (0.0281)
			parametrical	0.9447	0.3979 (0.0561)
		20.00%	BC_a bootstrap	0.2685	0.6344 (0.0410)
			parametrical	0.8525	1.0466 (0.1329)
		50.00%	BC_a bootstrap	0.0000	0.7402 (0.0219)
			parametrical	0.0467	1.4295 (0.0773)
		65.54%	BC_a bootstrap	0.0000	0.7103 (0.0316)
			parametrical	0.0000	1.3121 (0.1135)
exponential	censored sample method	0.00%	BC_a bootstrap	0.9425	4.1493 (0.6068)
			parametrical	0.9535	4.2089 (0.4193)
		20.00%	BC_a bootstrap	0.9427	4.1500 (0.6045)
			parametrical	0.9538	4.2090 (0.4195)
		50.00%	BC_a bootstrap	0.9438	4.1949 (0.6037)
			parametrical	0.9520	4.2068 (0.4242)
		65.54%	BC_a bootstrap	0.9415	4.2921 (0.6104)
			parametrical	0.9449	4.2046 (0.4335)
	simple imputation method	0.00%	BC_a bootstrap	0.9435	4.1491 (0.6051)
			parametrical	0.9535	4.2089 (0.4193)
		20.00%	BC_a bootstrap	0.9436	4.1449 (0.6050)
			parametrical	0.9536	4.2125 (0.4188)
		50.00%	BC_a bootstrap	0.9398	4.0607 (0.6160)
			parametrical	0.9531	4.2915 (0.4099)
		65.54%	BC_a bootstrap	0.8824	3.9039 (0.6324)
			parametrical	0.9318	4.4640 (0.3928)
Poisson	censored sample method	0.00%	BC_a bootstrap	0.9425	0.7792 (0.0600)
			parametrical	0.9493	0.7841 (0.0198)
		23.81%	BC_a bootstrap	0.9447	0.7817 (0.0595)
			parametrical	0.9469	0.7841 (0.0198)
		43.35%	BC_a bootstrap	0.9431	0.7912 (0.0583)
			parametrical	0.9418	0.7840 (0.0200)
		62.88%	BC_a bootstrap	0.9458	0.8176 (0.0573)
			parametrical	0.9360	0.7838 (0.0207)
	simple imputation method	0.00%	BC_a bootstrap	0.9467	0.7793 (0.0599)
			parametrical	0.9493	0.7841 (0.0198)
		23.81%	BC_a bootstrap	0.9476	0.7667 (0.0587)
			parametrical	0.9535	0.7859 (0.0194)
		43.35%	BC_a bootstrap	0.9445	0.7753 (0.0579)
			parametrical	0.9449	0.7832 (0.0197)
		62.88%	BC_a bootstrap	0.9245	0.7791 (0.0567)
			parametrical	0.9196	0.7756 (0.0200)

CI: confidence interval, prop.: proportion, sd: standard deviation. Simulation runs $B = 5500$, bootstrap replication number $Rb = 5500$, sample size was $N = 100$, 1 LLOQ.

Table C: **Evaluation of confidence intervals for 2 LLOQs, N=40.**

distribution	estimation method	censored prop.	CI type	coverage prop.	mean width of CI (sd)
normal	censored sample method	0.00	BC_a bootstrap	0.9404	0.6103 (0.0686)
			parametrical	0.9511	0.6368 (0.0712)
		20.00	BC_a bootstrap	0.9427	0.6451 (0.0977)
			parametrical	0.9436	0.6316 (0.0831)
		50.00	BC_a bootstrap	0.9427	0.8237 (0.2146)
			parametrical	0.8980	0.6324 (0.1131)
		65.54	BC_a bootstrap	0.9520	1.1069 (0.4002)
			parametrical	0.8011	0.6329 (0.1392)
	simple imputation method	0.00	BC_a bootstrap	0.9411	0.6106 (0.0689)
			parametrical	0.9387	0.6419 (0.1430)
		20.00	BC_a bootstrap	0.6198	0.9852 (0.0963)
			parametrical	0.9822	1.6491 (0.3240)
		50.00	BC_a bootstrap	0.0540	1.1516 (0.0553)
			parametrical	0.6209	2.2671 (0.2110)
		65.54	BC_a bootstrap	0.0036	1.1050 (0.0766)
			parametrical	0.1900	2.0686 (0.2921)
exponential	censored sample method	0.00	BC_a bootstrap	0.9264	6.5413 (1.5584)
			parametrical	0.9465	6.8456 (1.1028)
		20.00	BC_a bootstrap	0.9204	7.1022 (1.6185)
			parametrical	0.9207	6.9757 (1.2140)
		50.00	BC_a bootstrap	0.9291	6.7456 (1.6100)
			parametrical	0.9462	6.8388 (1.1173)
		65.54	BC_a bootstrap	0.9340	6.8645 (1.5769)
			parametrical	0.9369	6.8252 (1.1416)
	simple imputation method	0.00	BC_a bootstrap	0.9282	6.5380 (1.5524)
			parametrical	0.9465	6.8450 (1.1029)
		20.00	BC_a bootstrap	0.9282	6.5309 (1.5575)
			parametrical	0.9469	6.8553 (1.1010)
		50.00	BC_a bootstrap	0.9289	6.3847 (1.5901)
			parametrical	0.9556	7.0174 (1.0689)
		65.54	BC_a bootstrap	0.8962	6.1239 (1.6342)
			parametrical	0.9475	7.3616 (1.0143)
Poisson	censored sample method	0.00	BC_a bootstrap	0.9315	1.2175 (0.1467)
			parametrical	0.9418	1.2384 (0.0496)
		33.58	BC_a bootstrap	0.8793	1.5658 (0.3059)
			parametrical	0.8687	1.2512 (0.0630)
		53.11	BC_a bootstrap	0.9496	1.5702 (0.4752)
			parametrical	0.9311	1.2386 (0.0538)
		70.70	BC_a bootstrap	0.9436	1.3573 (0.2308)
			parametrical	0.9253	1.2368 (0.0542)
	simple imputation method	0.00	BC_a bootstrap	0.9373	1.2175 (0.1469)
			parametrical	0.9418	1.2384 (0.0496)
		33.58	BC_a bootstrap	0.9378	1.2057 (0.1432)
			parametrical	0.9451	1.2392 (0.0489)
		53.11	BC_a bootstrap	0.9367	1.2166 (0.1406)
			parametrical	0.9365	1.2310 (0.0496)
		70.70	BC_a bootstrap	0.9251	1.1966 (0.1442)
			parametrical	0.9244	1.2194 (0.0492)

CI: confidence interval, prop.: proportion, sd: standard deviation. Simulation runs $B = 5500$, bootstrap replication number $Rb = 5500$, sample size was $N = 40$, 2 LLOQ.

Table D: **Evaluation of confidence intervals for 1 LLOQ, N=40.**

distribution	estimation method	censored prop.	CI type	coverage prop.	mean width of CI (sd)
normal	censored sample method	0.00	BC_a bootstrap	0.9415	0.6102 (0.0685)
normal	censored sample method	0.00	parametrical	0.9511	0.6368 (0.0712)
normal	censored sample method	20.00	BC_a bootstrap	0.9473	0.6479 (0.0986)
normal	censored sample method	20.00	parametrical	0.9460	0.6333 (0.0834)
normal	censored sample method	50.00	BC_a bootstrap	0.9402	0.8262 (0.2201)
normal	censored sample method	50.00	parametrical	0.8984	0.6329 (0.1144)
normal	censored sample method	65.54	BC_a bootstrap	0.9440	1.1155 (0.4273)
normal	censored sample method	65.54	parametrical	0.7951	0.6308 (0.1448)
normal	simple imputation method	0.00	BC_a bootstrap	0.9411	0.6103 (0.0687)
normal	simple imputation method	0.00	parametrical	0.9387	0.6419 (0.1430)
normal	simple imputation method	20.00	BC_a bootstrap	0.6247	0.9969 (0.1006)
normal	simple imputation method	20.00	parametrical	0.9878	1.6879 (0.3416)
normal	simple imputation method	50.00	BC_a bootstrap	0.0533	1.1619 (0.0535)
normal	simple imputation method	50.00	parametrical	0.6453	2.3086 (0.2060)
normal	simple imputation method	65.54	BC_a bootstrap	0.0042	1.1192 (0.0762)
normal	simple imputation method	65.54	parametrical	0.2125	2.1220 (0.2951)
exponential	censored sample method	0.00	BC_a bootstrap	0.9280	6.5417 (1.5560)
exponential	censored sample method	0.00	parametrical	0.9465	6.8450 (1.1029)
exponential	censored sample method	20.00	BC_a bootstrap	0.9273	6.5423 (1.5577)
exponential	censored sample method	20.00	parametrical	0.9467	6.8448 (1.1035)
exponential	censored sample method	50.00	BC_a bootstrap	0.9251	6.6311 (1.5553)
exponential	censored sample method	50.00	parametrical	0.9420	6.8360 (1.1146)
exponential	censored sample method	65.54	BC_a bootstrap	0.9302	6.8043 (1.5629)
exponential	censored sample method	65.54	parametrical	0.9395	6.8283 (1.1362)
exponential	simple imputation method	0.00	BC_a bootstrap	0.9289	6.5418 (1.5551)
exponential	simple imputation method	0.00	parametrical	0.9465	6.8450 (1.1029)
exponential	simple imputation method	20.00	BC_a bootstrap	0.9295	6.5351 (1.5590)
exponential	simple imputation method	20.00	parametrical	0.9478	6.8507 (1.1016)
exponential	simple imputation method	50.00	BC_a bootstrap	0.9276	6.4109 (1.5783)
exponential	simple imputation method	50.00	parametrical	0.9533	6.9778 (1.0760)
exponential	simple imputation method	65.54	BC_a bootstrap	0.9127	6.1844 (1.6313)
exponential	simple imputation method	65.54	parametrical	0.9542	7.2610 (1.0280)
Poisson	censored sample method	0.00	BC_a bootstrap	0.9302	1.2169 (0.1470)
Poisson	censored sample method	0.00	parametrical	0.9418	1.2384 (0.0496)
Poisson	censored sample method	23.81	BC_a bootstrap	0.9364	1.2229 (0.1467)
Poisson	censored sample method	23.81	parametrical	0.9431	1.2383 (0.0497)
Poisson	censored sample method	43.35	BC_a bootstrap	0.9358	1.2409 (0.1442)
Poisson	censored sample method	43.35	parametrical	0.9395	1.2380 (0.0503)
Poisson	censored sample method	62.88	BC_a bootstrap	0.9424	1.2883 (0.1406)
Poisson	censored sample method	62.88	parametrical	0.9324	1.2372 (0.0522)
Poisson	simple imputation method	0.00	BC_a bootstrap	0.9387	1.2169 (0.1472)
Poisson	simple imputation method	0.00	parametrical	0.9418	1.2384 (0.0496)
Poisson	simple imputation method	23.81	BC_a bootstrap	0.9411	1.1975 (0.1442)
Poisson	simple imputation method	23.81	parametrical	0.9453	1.2413 (0.0487)
Poisson	simple imputation method	43.35	BC_a bootstrap	0.9391	1.2122 (0.1420)
Poisson	simple imputation method	43.35	parametrical	0.9409	1.2370 (0.0493)
Poisson	simple imputation method	62.88	BC_a bootstrap	0.9335	1.2175 (0.1390)
Poisson	simple imputation method	62.88	parametrical	0.9265	1.2250 (0.0501)

CI: confidence interval, prop.: proportion, sd: standard deviation. Simulation runs $B = 5500$, bootstrap replication number $Rb = 5500$, sample size was $N = 40$, 1 LLOQ.
