# Supplementary material for: Confidence interval comparison: Precision of maximum likelihood estimates in LLOQ affected data
Source: PLoS One. 2023 Nov 2;18(11):e0293640. doi: 10.1371/journal.pone.0293640 (PMC10621850; doi:10.1371/journal.pone.0293640)
Supplement: S5 File — Corresponding Figures for the confidence interval asessment under distributional misspecification for normal and Poisson distribution for a sample size of N = 100 and two LLOQs. (PDF) [file pone.0293640.s006.pdf]

S5 File

Evaluation of confidence intervals under distributional misspecification through coverage proportion and width of CI.

For normal assumption, N=100, 2 LLOQ.

For Poisson assumption, N=100, 2 LLOQ.

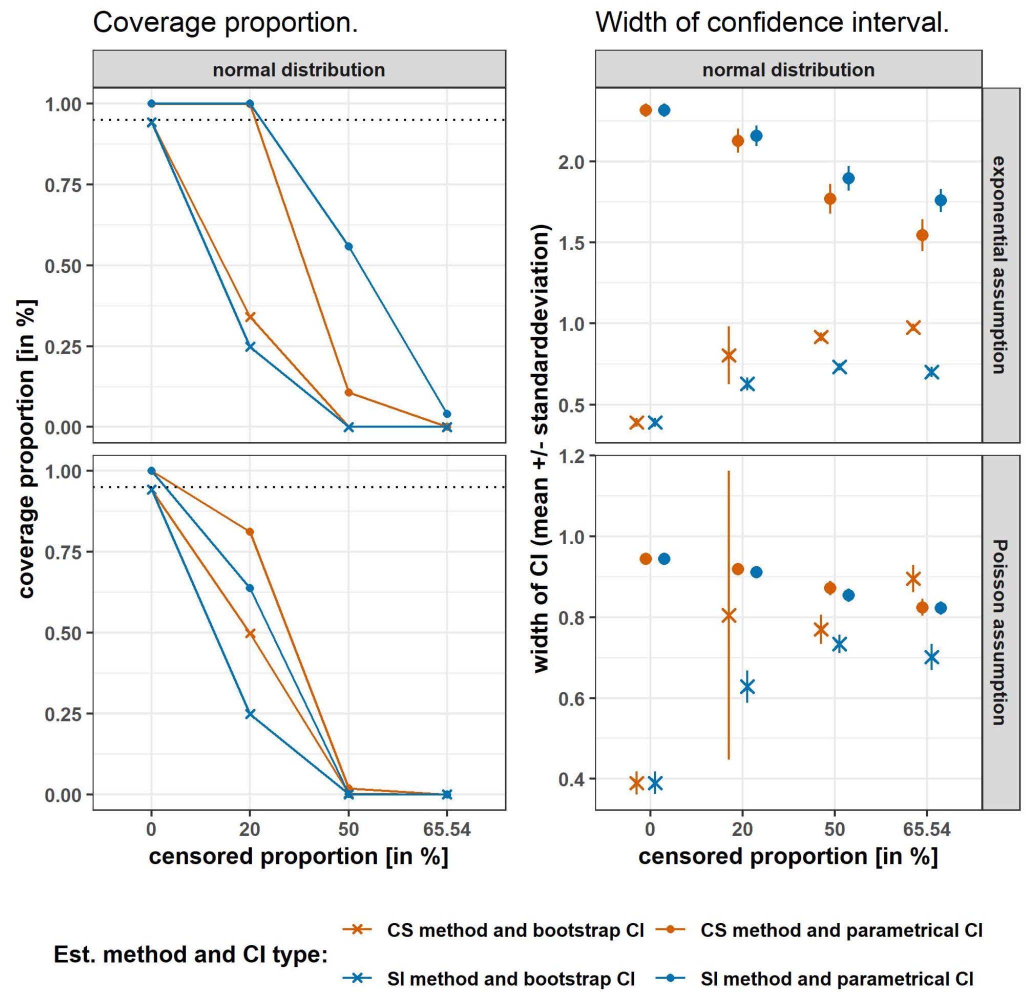

Figure A: **Under assumptional misspecification simulated coverage proportion and mean width of confidence intervals for normally distributed data for 2 LLOQs.** Performance of both point estimation methods combined with both CIs for normally distributed data under respective assumptional misspecification, meaning exponential assumption and Poisson assumption. The results of the censored sample method (CS) in orange versus simple imputation method (SI) in blue in the scenario of two LLOQs present, results of the BC_a bootstrap CI marked with an x and of the parametrical CI with a dot. Coverage proportion is shown in the left hand side and width of the CI with mean and standarddeviation on the rigth hand side. Different censored proportions are shown on the x-axis. As a dotted line on the left hand side, the theoretically aimed coverage proportion of 95% is presented, indicating estimates closer to the dotted line as better. For $B = 5500$, $Rb = 5500$, and $N = 100$.

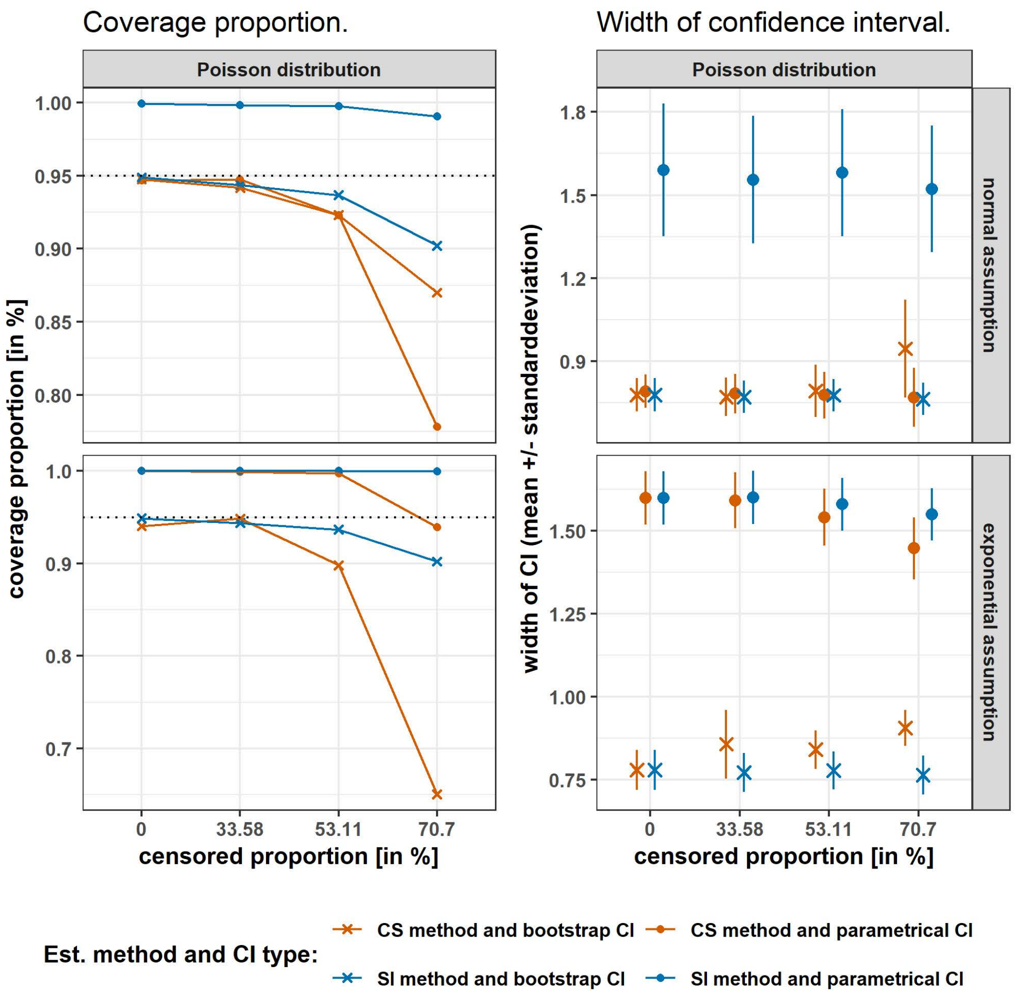

Figure B: **Under assumptional misspecification simulated coverage proportion and mean width of confidence intervals for Poisson distributed data for 2 LLOQs.** Performance of both point estimation methods combined with both CIs for Poisson distributed data under respective assumptional misspecification, meaning normal assumption and exponential assumption. The results of the censored sample method (CS) in orange versus simple imputation method (SI) in blue in the scenario of two LLOQs present, results of the BC_a bootstrap CI marked with an x and of the parametrical CI with a dot. Coverage proportion is shown in the left hand side and width of the CI with mean and standarddeviation on the rigth hand side. Different censored proportions are shown on the x-axis. As a dotted line on the left hand side, the theoretically aimed coverage proportion of 95% is presented, indicating estimates closer to the dotted line as better. For $B = 5500$, $Rb = 5500$, and $N = 100$.
